# Computational model predicts protein binding sites of a luminescent ligand equipped with guanidiniocarbonyl-pyrrole groups

**DOI:** 10.3762/bjoc.18.137

**Published:** 2022-09-23

**Authors:** Neda Rafieiolhosseini, Matthias Killa, Thorben Neumann, Niklas Tötsch, Jean-Noël Grad, Alexander Höing, Thies Dirksmeyer, Jochen Niemeyer, Christian Ottmann, Shirley K Knauer, Michael Giese, Jens Voskuhl, Daniel Hoffmann

**Affiliations:** 1 Bioinformatics and Computational Biophysics, Center for Medical Biotechnology (ZMB), University of Duisburg-Essen, Universitätsstraße 5, 45141 Essen, Germany; 2 Faculty of Chemistry (Organic Chemistry) and CENIDE, University of Duisburg-Essen, Universitätsstraße 7, 45141 Essen, Germany; 3 Institute for Computational Physics, University of Stuttgart, Allmandring 3, 70569 Stuttgart, Germany; 4 Department of Molecular Biology II, Center for Medical Biotechnology (ZMB), University of Duisburg-Essen, Universitätsstraße 5, 45141 Essen, Germany; 5 Laboratory of Chemical Biology, Department of Biomedical Engineering and Institute for Complex Molecular Systems, Eindhoven University of Technology, PO Box 513, 5600 MB Eindhoven, Netherlands

**Keywords:** AIE luminophores, fluorescence emission, guanidiniocarbonyl-pyrrole, ligand binding, 14-3-3 protein

## Abstract

The 14-3-3 protein family, one of the first discovered phosphoserine/phosphothreonine binding proteins, has attracted interest not only because of its important role in the cell regulatory processes but also due to its enormous number of interactions with other proteins. Here, we use a computational approach to predict the binding sites of the designed hybrid compound featuring aggregation-induced emission luminophores as a potential supramolecular ligand for 14-3-3ζ in the presence and absence of C-Raf peptides. Our results suggest that the area above and below the central pore of the dimeric 14-3-3ζ protein is the most probable binding site for the ligand. Moreover, we predict that the position of the ligand is sensitive to the presence of phosphorylated C-Raf peptides. With a series of experiments, we confirmed the computational prediction of two *C*_2_ related, dominating binding sites on 14-3-3ζ that may bind to two of the supramolecular ligand molecules.

## Introduction

The 14-3-3 protein family was one of the first discovered phosphoserine/phosphothreonine binding proteins. In total seven isoforms of the 14-3-3 family are known to date (β, ε, γ, τ, θ, σ, and ζ) in mammals [1]. They are forming homo and heterodimers with a profile shaped like the Greek letter “ω” [2]. 14-3-3 proteins have attracted interest due to their enormous number of interactions with other proteins. Currently, more than 200 interacting proteins are known. 14-3-3 proteins play a significant role in cell signaling [2–3] and they were found to be essential in processes such as differentiation, apoptosis, or migration [4]. 14-3-3 proteins also play a role in several human diseases including cancer and neurodegenerative disorders like Alzheimer’s and Parkinson’s diseases [5]. Based on these observations and findings it is not surprising that the 14-3-3 family has attracted interest in pharmacological research as a novel potential target [5–6].

One option to modulate, inhibit, or stabilize protein–protein interactions (PPI) is the use of specific supramolecular ligands [7–8]. One well-known example of efficient protein binders is the so-called guanidiniocarbonyl-pyrrole (GCP) developed 20 years ago by Schmuck et al*.* [9]. These compounds are known to efficiently bind to oxo-anions such as carboxylates [10]. These compounds were already used to specifically address carboxylates on the surface of proteins. Many artificial receptors based on guanidinium scaffolds use hydrogen bonding, charge pairing, and hydrophobic interactions to complex oxo-anions [11]. The guanidiniocarbonyl-pyrrole (GCP) is able to bind oxo-anions even in aqueous solvents with competing ions and salts. Schmuck et al. also discovered that an additional positive charge increases the binding affinity to oxo-anions [10]. These unique properties make the GCP oxo-anion binder an ideal candidate to be used for protein recognition. In a previous study, GCP containing polycationic ligands for 14-3-3 proteins had a significant effect on PPIs [12–13]. Furthermore, a simple GCP derivative, GCP-Lys-OMe, was identified as the first binder for the specific binding area of the 14-3-3ζ homodimer [14]. Very recently the survivin–histone H3 interaction was disrupted using a GCP dimer, which led to decreased cancer cell proliferation [8].

A major problem in this context is the readout of binding events, which is currently mainly achieved by indirect measurements. One approach to overcome this issue is to use fluorescence emission as a read-out tool, such as an emission “on” or “off” behavior [15]. Selective and sensitive fluorescent ligands have been proven to be essential tools for the study of biological systems by biosensing and imaging [16]. There is an increasing demand for novel luminophores tailor-made for different applications. One unique, promising class of compounds, the so-called aggregation-induced emitters (AIE) have been used for a wide range of applications, e.g., in OLEDs, liquid crystals [17], stimuli responses, bioassays, protein and ion detection or imaging [15,18]. In contrast to classical luminophores, these compounds typically show emission “on” behavior upon aggregation or binding, which can be explained by a restriction of motion. In this contribution, we designed a hybrid compound featuring AIE luminophores based on aromatic thioethers [19] as a potential supramolecular ligand for 14-3-3ζ.

**Figure 1:**
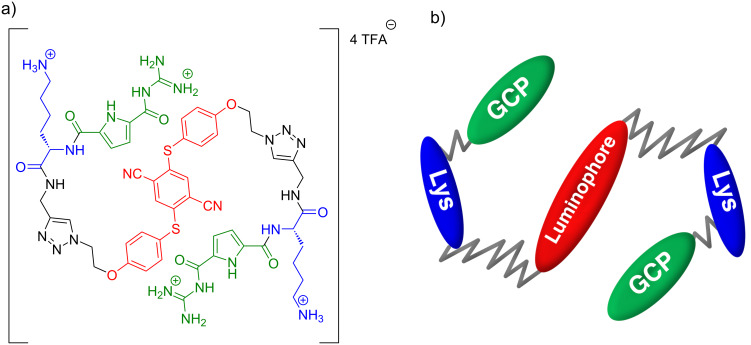
AIE-active molecule **1**. (a) Structure of **1** with color-coded subunits AIE, lysine, and GCP. (b) Coarse-grained bead-spring model of **1**.

**Figure 2:**
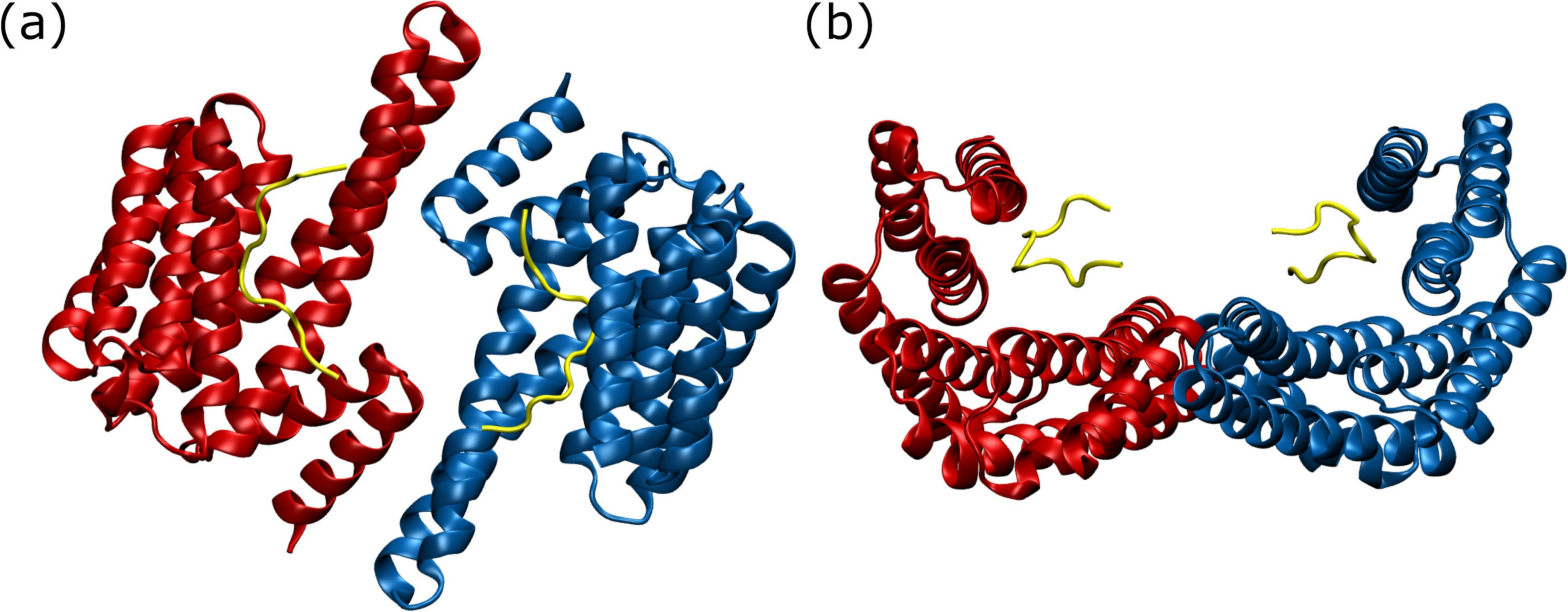
14-3-3ζ from (a) top and (b) side with the two monomers in red and blue. In the top view the central pore is clearly visible. The side view shows the ω shape with the two binding grooves for protein ligands, e.g., the C-Raf peptides (yellow). The structure is based on PDB entry 4IHL [20].

We synthesized a GCP-Lys dimer coupled via Cu(I)-catalyzed click reaction to the chosen emitter equipped with two azide functions (Figure 1 and Supporting Information File 1) and investigated the photophysical properties in detail (Supporting Information File 1, Figures S22–S25). This compound **1** was tested in initial binding assays using fluorescence emission as well as native gel electrophoresis. We could indeed show that **1** binds to 14-3-3ζ as detected by native gel electrophoresis and fluorescence titration (Supporting Information File 1). Experiments show that probably two molecules of **1** can bind simultaneously to one 14-3-3ζ homodimer (Supporting Information File 1, Figure S29). However, it is completely unknown where **1** binds on the 14-3-3ζ’s surface. In its profile, 14-3-3ζ has roughly the shape of the Greek letter ω, where the two wells can clamp together pairs of proteins (Figure 2b). Knowledge of the binding site is of critical importance for a potential bioanalytics application: if **1** would block one or both wells of the ω, it could prevent binding of ligand proteins, whereas a **1** binding site in the outer parts of the ω would not interfere with functional protein–protein binding.

One theoretical option to identify putative binding sites of compound **1** on 14-3-3ζ would be by all-atom molecular dynamics (MD) simulations. However, as numerous studies have shown [21–23], the resources needed for a suitable sampling of such flexible systems by MD are typically underestimated by orders of magnitude and not accessible to many researchers. A conceivable alternative approach would be the use of docking software such as Autodock Vina [24–25]. However, these docking methods typically have been developed to dock ligands that are not too large and not too flexible and ideally have well-defined binding pockets. In contrast, our ligand **1** is large and flexible and does not bind into well-defined pockets but likely is loosely bound to the protein surface by charge–charge interactions. Therefore, standard docking methods are not an option. Since this class of large, flexible, charged ligands is biologically important, we have previously devised a method, Epitopsy [26], to identify binding sites of fragments of such ligands on proteins. In this work we go a significant step further: to identify the putative binding sites of the full compound **1** on 14-3-3ζ, and possibly to guide further experiments, we, first, developed a computational model of the molecular system consisting of 14-3-3ζ, **1**, and an implicit solvent. Second, we devised a new method that allowed a complete screening of the modeled system at a meaningful level of accuracy and that identified putative binding sites of **1** on 14-3-3ζ. These predictions can then be tested in a targeted way. Using this approach, we tried to answer basically two questions: (a) where does compound **1** bind on 14-3-3ζ, and (b) how would the presence of phosphorylated C-Raf peptides in the binding grooves of 14-3-3ζ affect the binding of **1**?

## Results and Discussion

**Figure 3:**
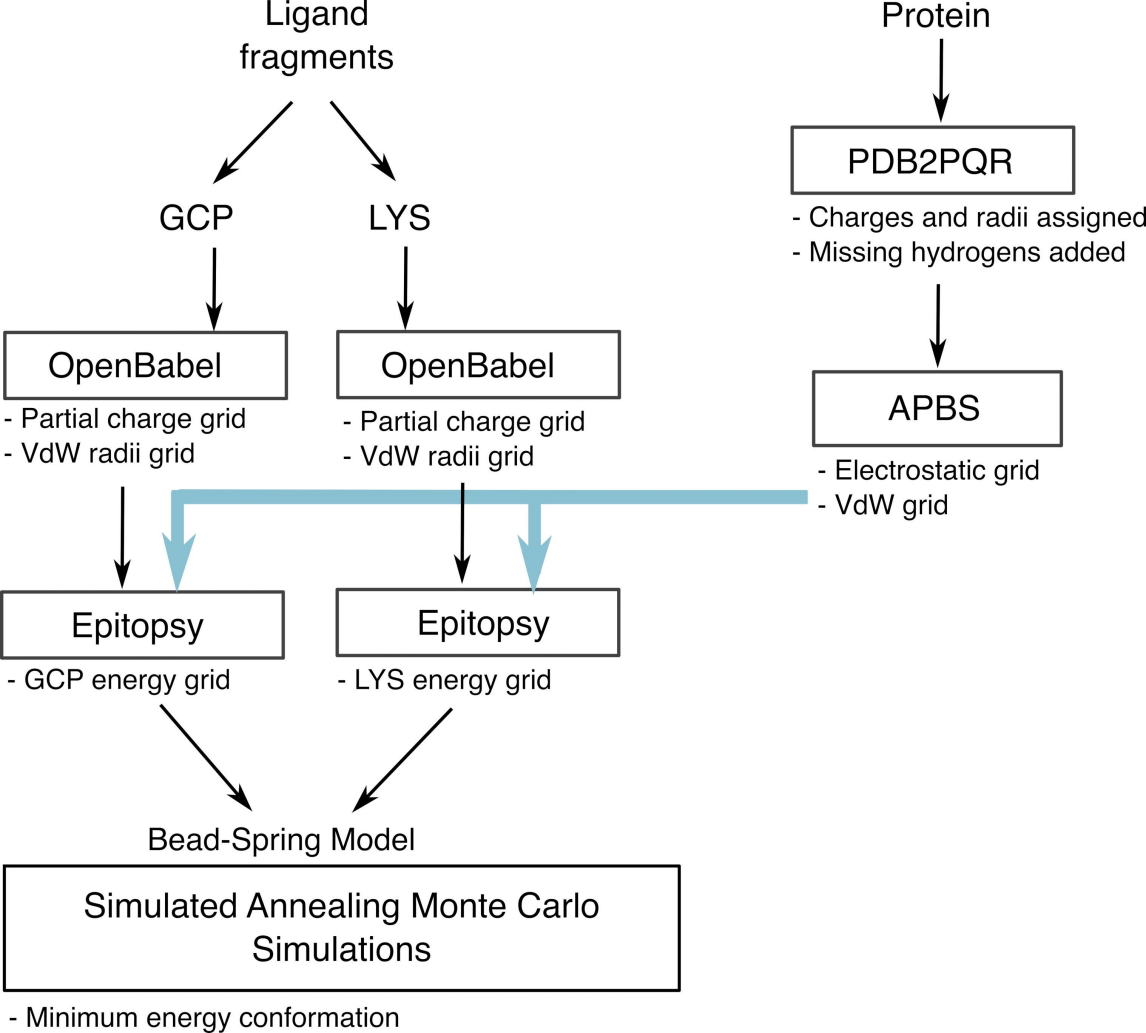
Workflow of the computational approach used in this study. The protein structure and the structure of the ligand fragments are the required inputs. Charges and radii are assigned to both interaction partners in the represented way. A map of affinities of protein with each of the two ligand fragments is obtained using Epitopsy. Our Simulated Annealing Monte Carlo simulations read the interaction potentials from the energy grids obtained from Epitopsy. The results of our simulations are then analyzed to find the minimum energy conformation which is the most probable binding position of the ligand.

We explored potential binding positions of **1** around 14-3-3ζ with and without C-Raf peptides by exhaustive simulations. Figure 3 represents the overall workflow of our computational approach which is described in detail in the Experimental section of this article.

**Figure 4:**
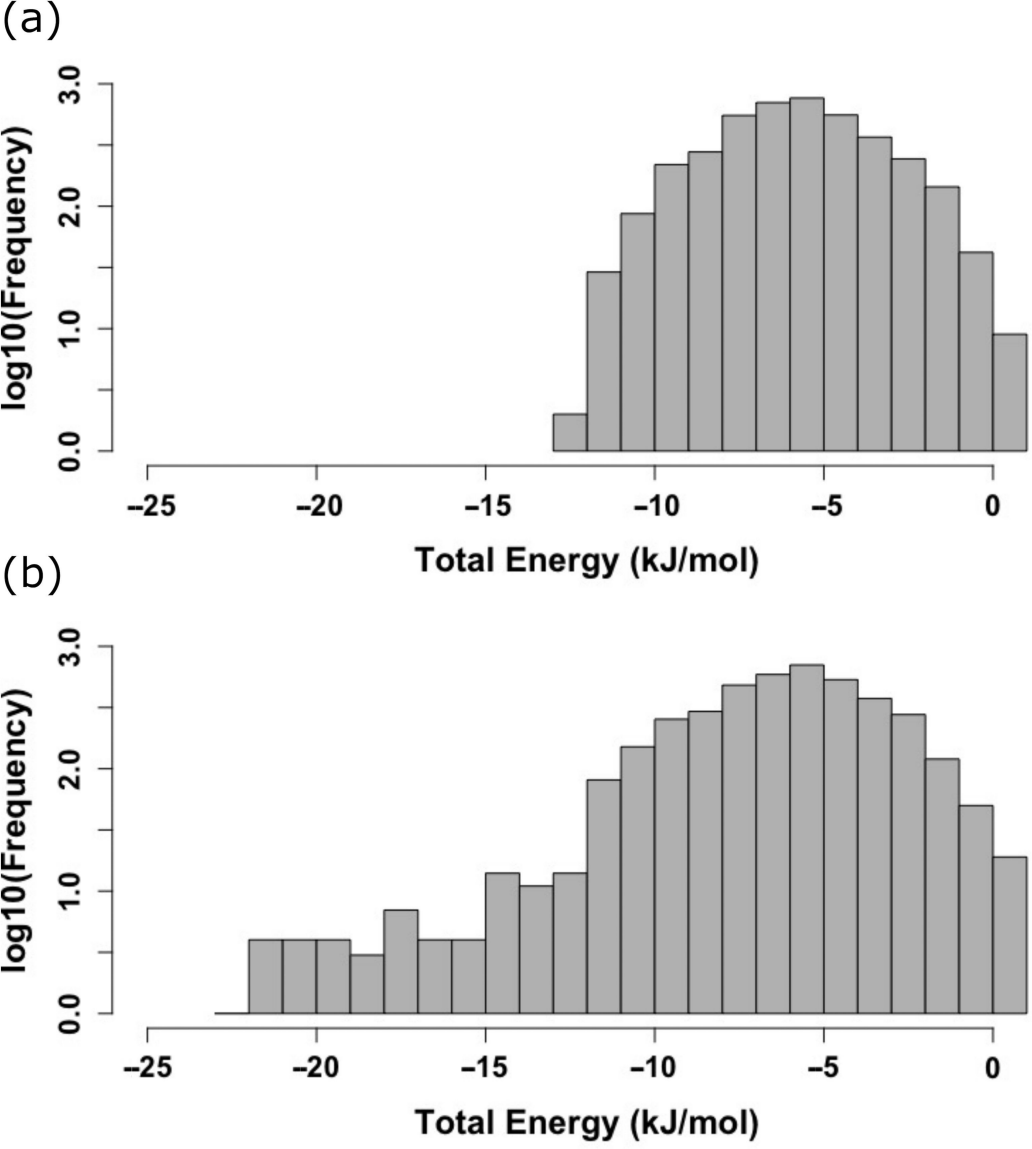
Log-scaled histogram of total energies at the final steps of all simulations. (a) Simulated annealing (SA) runs with **1** around 14-3-3ζ protein, (b) **1** around 14-3-3ζ/C-Raf complex.

The distribution of the final total energies is shown in Figure 4. Although the bulk of the energies follows a similar distribution in both simulation series, there is a remarkable difference, namely that in the simulations with C-Raf the log-scaled histogram has a tail to much lower energies. This difference must be due to interactions of **1** with C-Raf because otherwise the molecular systems are unchanged.

### Predicted binding sites of 1 on 14-3-3ζ

**Figure 5:**
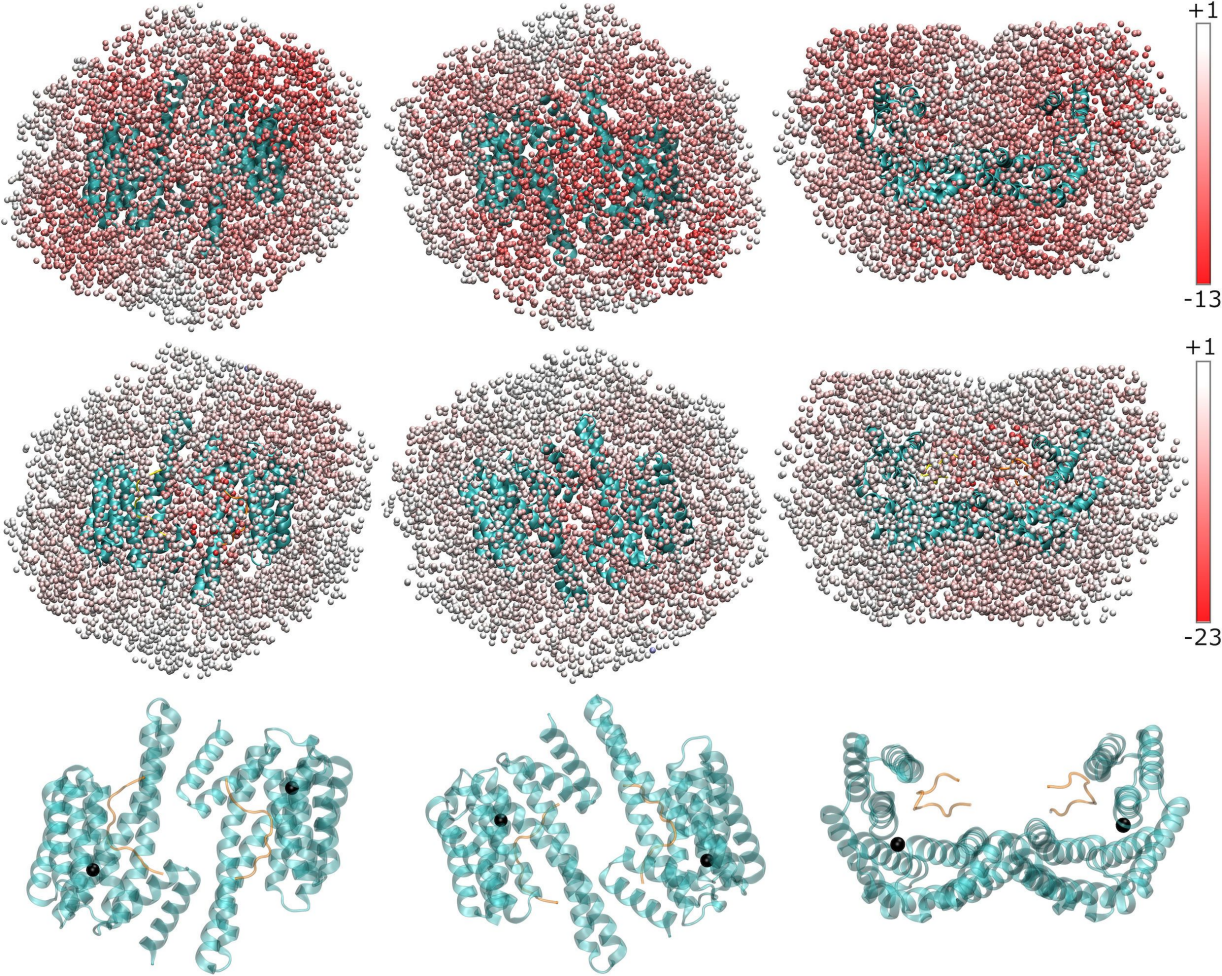
Sampled positions of the AIE moiety colored according to the total energy of **1** from dark red (lowest energy) to white (highest energy). The values in the color bar are in units of kJ/mol. The three columns correspond to three different perspectives (top view in left column, bottom view in central column, side view in right column). Top row: in absence of C-Raf; middle row: in presence of C-Raf; bottom row: structure of 14-3-3ζ in corresponding orientations (same orientation in each column). Little black spheres in the bottom row are cluster medoids of the ligand positions.

The total energies at the final positions of simulated annealing (SA) runs (Figure 5) show that **1** could bind in most regions on the surface of 14-3-3ζ, especially in the absence of C-Raf (top row in Figure 5). Some areas seem to be favored: firstly, the tips of the omega ears, though this should be taken with caution as the model is lacking the very flexible C-termini that are in these regions; secondly, below the pore at the bottom of the omega in the case without C-Raf; thirdly, above the pore between the two binding grooves of the omega in the presence of C-Raf. Overall, the clustering and the pattern of energies reflect the *C*_2_ symmetry of the 14-3-3ζ dimer.

### Minimum energy conformations

**Figure 6:**
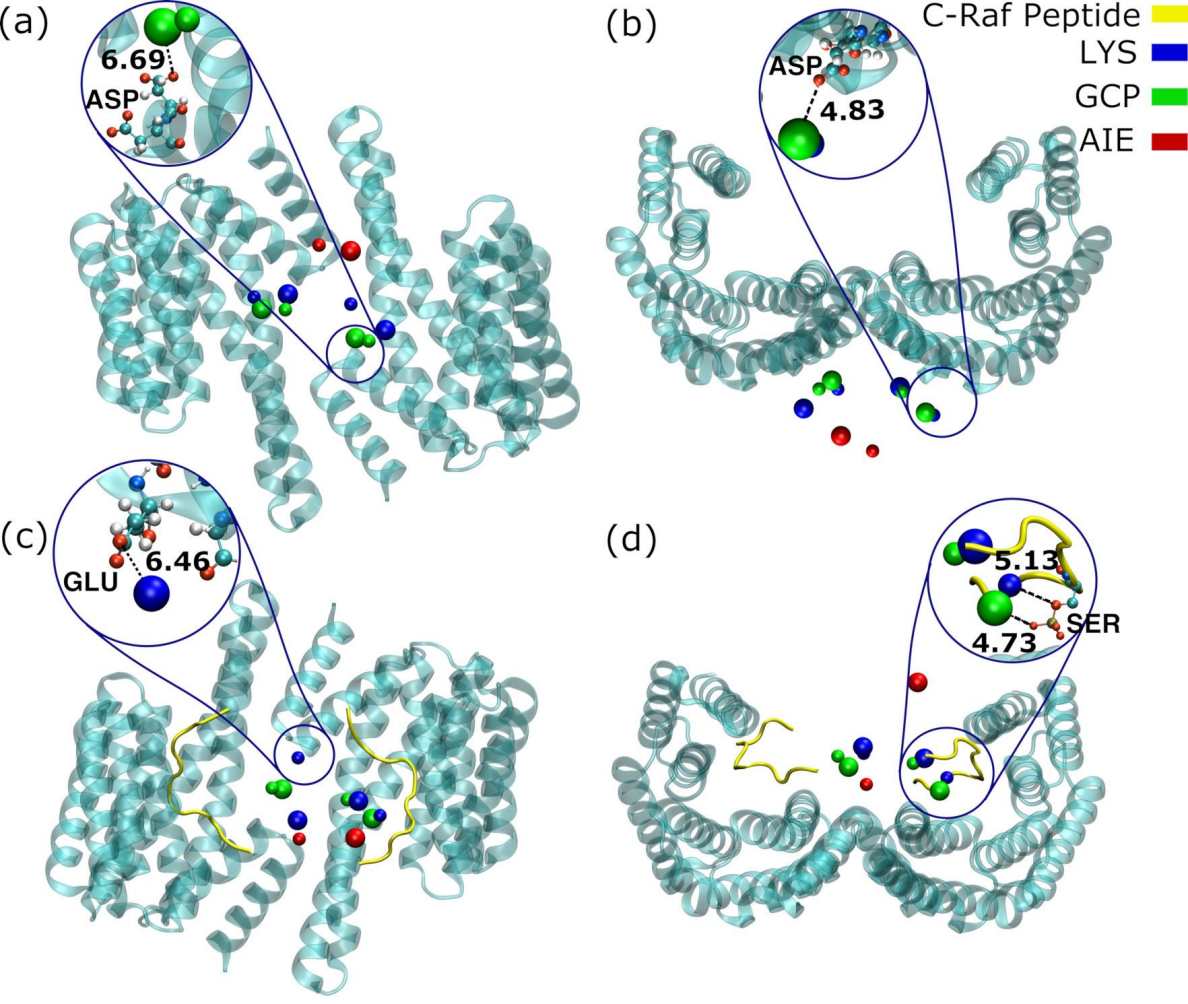
Minimum energy conformations of AIE ligand in the absence (a,b) and presence (c,d) of C-Raf peptides (bottom view in (a), side view in (b) and (d), top view in (c)). The first and second minimum energy conformations are represented by small and large beads, respectively. In the absence of the C-Raf peptides, Lys (dark blue beads) and GCP (green beads) groups are found near the aspartic (ASP) and glutamic acid (GLU) residues of 14-3-3ζ. In the presence of the peptides, at least one Lys or GCP group is found near the phosphorylated serine (SER) group (magnified image in (d)) of the C-Raf peptides. In this figure, all the distances are in 0.1 nm.

Closer inspection of energies reveals that in the absence of C-Raf peptides, the minimum energy conformation of **1** lies below the ω, under the central pore of 14-3-3ζ (Figure 6a and b). With this conformation, one of the GCP groups falls into the energy minimum of the GCP affinity grid. The same happens to the Lys groups. As Figure 6 shows, the GCP and lysine beads are found near the hydroxyl groups of the aspartic acid residues of the protein. The second energy minimum of the ligand is related to the first one by the *C_2_* symmetry of 14-3-3ζ.

In contrast, in the presence of C-Raf peptides the minimum energy positions of **1** move to the center of the ω where the AIE moieties hover above the central pore (Figure 6c and d). In these two conformations, at least one of the lysine groups lies at the minimum energy position of the lysine affinity map (more information about the affinity map is given in the “Energy grids” section). Both GCP groups locate in the two minimum energy positions of the GCP affinity map as well. In both minimum energy conformations of the ligand, at least one GCP or Lys group is found near one of the phosphorylated serine (pSer) residues of the C-Raf peptides (Figure 6d). This is consistent with a strong interaction of the positive GCP and Lys groups with the highly negatively charged pSer.

### Binding experiments

A qualitative proof of binding of **1** and 14-3-3ζ could be achieved with native gel electrophoresis. It could be shown that **1** hinders some of the 14-3-3ζ from entering the gel and that the mobility of 14-3-3ζ decreases with the amount of **1** added. In fact, using default atomic charges [27–28], the 14-3-3ζ dimer has a net charge of −32 |e| (proton charges), and the binding of two **1** leads to a net charge of −24 |e| of the complex.

It is known that aggregation-induced emission is caused by the blocking of non-radiative decay pathways leading to an emission “on” phenomenon [15]. According to this expectation, we observed an increase in emission at ≈470 nm upon titration of **1** to 14-3-3ζ, in initial experiments, which motivated this contribution (Supporting Information File 1, Figure S27). Additionally, fluorescence- and UV-titration experiments suggested a dissociation constant in the low micromolar range (5.0 ± 0.9 µM/7.5 ± 1.1 µM, respectively) in buffered aqueous solution (25 mM HEPES, 150 mM NaCl, 10 mM MgCl_2_, 0.5 mM TCEP, pH 6.5) (Supporting Information File 1, Figures S26 and S28). Furthermore, we discovered that two ligands **1** simultaneously bind to one 14-3-3ζ dimer as determined by a fluorescence titration using the Job’s method (Supporting Information File 1, Figure S29 [29]). This experimental result is consistent with our computational prediction that **1** has two high-affinity binding positions (Figure 6a and b) that could lead to restricted ligand flexibility and hence the observed increase of emission.

A more detailed experimental validation will be the focus of an experimental study in the near future. Three variants of **1** will be synthesized to gain a deeper insight into the binding stoichiometry and affinity, as well as the structure–property relationship on the influence on protein functions.

## Conclusion

On the basis of the final energy values of 4000 runs with a simulated annealing approach, we find the area above and below the central pore of 14-3-3ζ protein to be the most probable binding sites for **1**. The position of the ligand is sensitive to the presence of phosphorylated C-Raf peptides as interaction with these phosphorylated peptides draws **1** with its positive GCP and Lys groups into the region between the two binding grooves of 14-3-3ζ with a much higher affinity. If presence of another ligand (like C-Raf) leads to rearrangement of binding of AIE-molecules around a target protein (like 14-3-3ζ), and thus to differences in fluorescence, we have a mechanism that can possibly be exploited in analytical applications.

## Experimental

### Energy grids

To compute a map of affinities of 14-3-3ζ with GCP and lysine ligands, we proceeded as follows. The GCP and lysine ligand geometries were calculated in OpenBabel v2.3.2 [30] starting from a SMILES string of each ligand. The van der Waals radii of ligand atoms were added automatically by OpenBabel. The 14-3-3ζ structure (PDB 4IHL, [20]) was refined with MODELLER [31] as described in reference [26]. Charges, van der Waals radii and missing hydrogen atoms were added by PDB2PQR v2.0.0 [27–28] at pH 6.5 with the Amber force field option (values are provided as a table in Supporting Information File 2 and in Supporting Information File 3, respectively). The 14-3-3ζ electrostatic field was calculated by solving the non-linear Poisson–Boltzmann equation with APBS version 1.5 [32] with ionic concentrations of 0.1 mol/L NaCl and 0.01 mol/L MgCl_2_ and relative dielectric permittivities ε_r_^protein^ = 2 and ε_r_^water^ = 79. To convert the electrostatic potential grid into energy grids suitable for the Hamiltonian, chemically relevant polymer fragments were used as molecular probes in Epitopsy [26]. Epitopsy is a tool designed to calculate the electrostatic energy of a protein–ligand system from the protein potential grid and ligand charge distribution; this approach captures ion-size effects and yields energy grids that are directly comparable to molecular dynamics simulation data. Accordingly, the 14-3-3ζ environment was scanned with the GCP and lysine ligands separately in Epitopsy using 150 rotations and a grid resolution of 0.4 Å to generate a pre-calculated Hamiltonian for a grid-based description of the electrostatic and van der Waals interactions. We have also tried a grid resolution of 0.8 Å which would have allowed more efficient calculations. However, the 0.8 Å resolution was not fine enough to accurately capture essential properties of the system, especially the *C*_2_ symmetry of 14-3-3ζ. Affinity grids for lysine and GCP have been obtained once using the structure of 4IHL (complex C-Raf/14-3-3ζ) and once using only the structure of 14-3-3ζ (4IHL without C-Raf peptides). These affinity grids are the only difference between the two series of simulations (in the presence and absence of C-Raf peptides).

It should be mentioned that Epitopsy does not model the displacement of structural water molecules and counter-ions when the ligand comes in contact with the protein surface and the ligand dielectric permittivity is approximately that of saline water. However, desolvation effects tend to be weak for solvent-exposed binding sites compared to binding pockets which are systematically avoided by Epitopsy due to the strong steric repulsion that is imposed on the ligand.

Note that the above approach models only interactions of individual GCP or lysine groups with 14-3-3ζ. To model the interactions of 14-3-3ζ with the full multivalent **1** ligand, we have combined these and further interactions in a single model as described in the following.

### Coarse-grained model

We developed a coarse-grained model of **1** known as the Bead-Spring model in the context of polymer studies [33–35]. Our model is a chain of five beads, GCP-Lys-AIE-Lys-GCP, with each pair of neighbors in the chain connected by a harmonic spring potential (Figure 1b). Given the symmetry of **1**, there are two types of springs, GCP-Lys and Lys-AIE. The spring parameters are chosen such that the five beads are always within a reasonable distance from each other, i.e., close to the distances in an atomistic model of the ligand structure. This atomistic model of the ligand was created in ChemDraw prime 16.0 (PerkinElmer, Waltham, MA, USA). The structure was then imported in Maestro (Schrödinger Maestro Version 11.5.011, MM share Version 4.1.011, Release 2018-1, Platform Windows-x64) and finally, the energy of the structure was minimized.

Overlaps between non-bonded beads are avoided by repelling potentials. Equation 1 represents this in the form of a hard sphere potential.


[1] $$E_{\text{repelling}}=\{\begin{array}{cc} \infty & d<a\\ 0 & d\geq a \end{array}$$

where *d* is the distance between the centers of two non-bonded beads and *a* is the average of pairwise addition of the radii of all the non-bonded beads (i.e., Lys-Lys, GCP-GCP, GCP-Lys, and GCP-AIE). According to the length scales in our atomistic model of the ligand, the value of 9.6 Å is predicted for *a*. To prevent possible overlaps between the ligand and the protein, atoms of the ligand are not allowed to enter the van der Waals radii of protein atoms. This model interacts with 14-3-3ζ through the energy grids of lysine and GCP as mentioned above. Equation 2 describes different terms of the total energy.


[2] $$E_{\text{total}}=\frac{1}{2}\sum_{i=1}^{4}k_{i}(l_{i}-l_{i}^{\text{eq}}{)}^{2}+E_{\text{g}}^{{\text{GCP}}_{1}}+E_{\text{g}}^{{\text{Lys}}_{1}}+E_{\text{g}}^{{\text{GCP}}_{\text{2}}}+E_{\text{g}}^{{\text{Lys}}_{\text{2}}}$$

where *k_i_* is the spring constant, *l_i_* is the distance between bonded beads *i* and *i+1*, and *l_i_*^eq^ is the corresponding distance at equilibrium, *E*_g_ represents the affinity of protein toward GCP or lysine at a specific grid point. In this study, *l*_1_ and *l*_4_, i.e. the equilibrium lengths for the springs connecting GCP to lysine, have been selected to be 6 Å and *l*_2_ and *l*_3_, i.e. the equilibrium lengths for the springs connecting lysine to AIE, have been selected to be 13 Å. The value of the spring constants in bead-spring models with Gaussian probability distribution is inversely proportional to the square of the equilibrium length of the spring [36]. Based on that, the values of *k*_1_ and *k*_4_ (spring constants for GCP-Lys bonds) were chosen to be 12 *k*_B_*T*/Å^2^ while the values of *k*_2_ and *k*_3_ (spring constants for Lys-AIE bonds) were chosen to be 3 *k*_B_*T*/Å^2^. With these parameters, the sum of harmonic potentials in the relaxed structure of the ligand will be comparable to the sum of GCP and lysine affinities (see Equation 2 for total potential). Otherwise, the minimized total energy might be misleading (the harmonic term might be minimized while the term related to the grid affinities might not be minimized). What matters is to find the minimum energy conformation of the ligand with regard to the affinity maps of lysine and GCP.

### Simulated annealing schedule

To identify the energy minima of the coarse-grained ligand model around 14-3-3ζ, we used simulated annealing. This computational method is typically applied to identify the global optimum of an objective function, even if there are many local optima [37–39].

At the heart of our implementation of the SA method we carried out Markov Chain Monte Carlo (MCMC) simulations of **1** around 14-3-3ζ with the acceptance criterion of Metropolis et al. [40], as detailed below. We did many MCMC simulations at temperatures given by the following schedule. We started the simulations at a very high temperature (3000 K) and slowly cooled the system down to low enough temperatures at which an acceptance ratio of less than 1% was achieved. At each temperature level, we ran an MCMC simulation with the Metropolis criterion. The Monte Carlo trajectory ended at each temperature level after 10000 moves, or after 1000 accepted moves, whichever was reached sooner. With this protocol good convergence to minimum energies was achieved (see Figure S5 in the Supplementary Information File 1).

We did 4000 SA runs to identify the globally optimal configuration of **1** around 14-3-3ζ. At the beginning of each simulation, we put the AIE bead at a random grid position within a volume layer around the protein (we used the same grid geometry as in the energy grids described above). The thickness of the layer corresponded to the distance between AIE and GCP. Its volume is about 500 nm^3^, so that the 4000 AIE positions sample the layer at a density of about 8 per nm^3^ (first and second rows in Figure 5). After its random placement, the position of AIE bead was kept fixed throughout the respective simulation. In each MCMC step, just one of the lysine or GCP beads was randomly selected and moved by a maximum of 6 grid lengths along each of the three Cartesian coordinate axes. If in the new position the bead fell within the specified distance of any of the non-bonded beads, the move was rejected due to the repelling potential. Otherwise, the energy with the new ligand position was evaluated and compared to the old energy in the Metropolis criterion to accept or reject the move. Our code was written in the Julia language and ran with Julia version 1.5.2 [41].

To validate our method, we applied it to the QQJ-096/14-3-3/c-Raf complex for which the potential binding site was available from all-atom molecular dynamics simulations in a previous study [26]. Our results for this system are reported in the first part of the Supporting Information File 1.

## Supporting Information

File 1 General information and instrumentation, cluster analysis, electrostatic potential surface of 14-3-3ζ, total energy of a simulation.

File 2 PDB2PQR output for lysine.

File 3 PDB2PQR output for GCP.
