# Supplementary material for: Computational model predicts protein binding sites of a luminescent ligand equipped with guanidiniocarbonyl-pyrrole groups
Source: Beilstein J Org Chem. 2022 Sep 23;18:1322–31. doi: 10.3762/bjoc.18.137 (PMC9520824; doi:10.3762/bjoc.18.137)
Supplement: File 1 — General information and instrumentation, cluster analysis, electrostatic potential surface of 14-3-3ζ, total energy of a simulation. [file Beilstein_J_Org_Chem-18-1322-s001.pdf]

BEILSTEIN JOURNAL OF ORGANIC CHEMISTRY

Supporting Information

for

Computational model predicts protein binding sites of a luminescent ligand equipped with guanidiniocarbonyl-pyrrole groups

Neda Rafieiolhosseini, Matthias Killa, Thorben Neumann, Niklas Tötsch, Jean-Noël Grad, Alexander Höing, Thies Dirksmeyer, Jochen Niemeyer, Christian Ottmann, Shirley K. Knauer, Michael Giese, Jens Voskuhl and Daniel Hoffmann

Beilstein J. Org. Chem. **2022,** *18,* 1322–1331. doi:10.3762/bjoc.18.137

General information and instrumentation, cluster analysis, electrostatic potential surface of 14-3-3ζ, total energy of a simulation

License and Terms: This is a supporting information file under the terms of the Creative Commons Attribution License (https://creativecommons.org/licenses/by/4.0). Please note that the reuse, redistribution and reproduction in particular requires that the author(s) and source are credited and that individual graphics may be subject to special legal provisions.
The license is subject to the *Beilstein Journal of Organic Chemistry* terms and conditions: (https://www.beilstein-journals.org/bjoc/terms)

Part 1: Applying SAMC approach to QQJ-096/14-3-3/c-Raf complex

To compare results of our SAMC method with much more expensive molecular dynamics simulations we use the complex of 14-3-3/c-Raf with the ligand QQJ-096. Binding sites of the supramolecular ligand QQJ-096 with 14-3-3/c-Raf complex have been investigated previously using molecular dynamics simulation and energy grid method [1]. Figure S1-A represents the structure of the QQJ-096 ligand with three arms, each containing two positively charged groups (a lysine and a GCP). The bead-spring model of this ligand that we use in this study is represented in Figure S1-B.

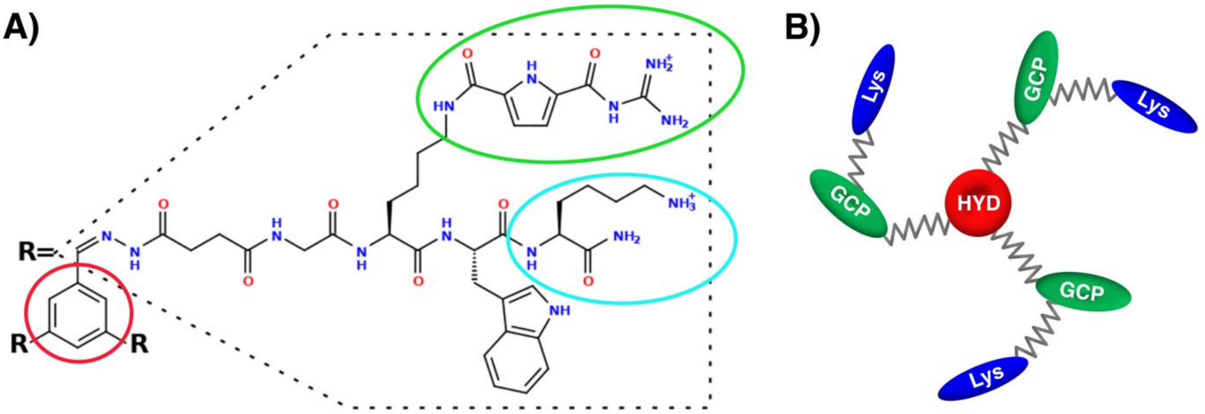

Figure S1: QQJ-096 ligand with only one of three arms depicted ("R"). A) Structure of QQJ-096 ligand. B) Coarse-grained bead-spring model of QQJ-096 ligand.

To estimate the spring parameters of the bead spring model of ligand QQJ-096, we used molecular dynamics trajectories of QQJ-096 in water from the previous study. We obtained the distance between GCP and lysine in each arm as a function of time by tracking the position of the ammonium atom in lysine and the central guanidinium carbon in GCP. Similarly, the

distance between GCP and HYD was obtained by tracking the position of C1 atom[1] in HYD and the central guanidinium carbon in GCP. We used these time series to calculate a probability distribution, whose logarithm gave us energy. Given a sufficiently long MD simulation of QQJ-096 in water, the three energy-distance plots (Figure S2) should be identical due to the threefold symmetry of the ligand. The difference we observe is attributed to under sampling. To improve the estimation of the two spring constants, we calculated the average of the three fitted polynomials which is a polynomial itself. For the spring connecting lysine and GCP we obtained an equilibrium length of 15 Å and a spring constant of 0.06 $k_B T/Å^2$. Similarly, for the spring connecting GCP and HYD we obtained an equilibrium length of 14 Å and a spring constant of 0.04 $k_B T/Å^2$.

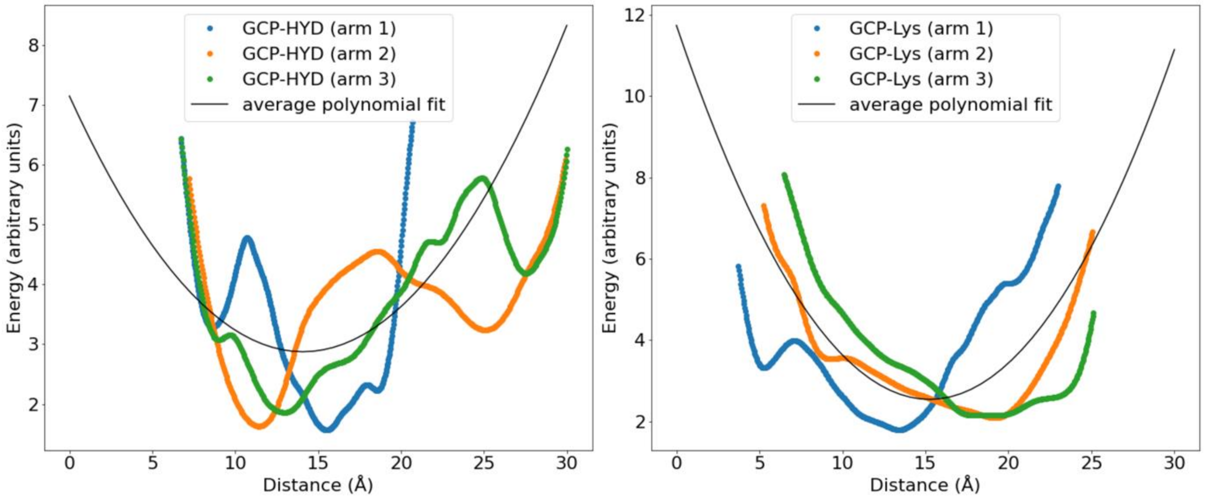

Figure S2: Energy vs distance plots for GCP-HYD group (left plot) and GCP-Lys group (right plot). The black curve represents the average of the three fitted polynomials.

We performed 4000 SAMC simulations on QQJ-096/14-3-3/c-Raf using the same protocol described in the “Experimental” section of the main manuscript. According to our results, both end groups show high affinity for the central cleft of 14-3-3/c-Raf (Figure S3, second and third rows) which is in line with the previously reported results [1]. In the previous study, the central

[1] Forcefield atom names follow the PDB nomenclature for the benzaldehyde ligand HBX (https://www.rcsb.org/ligand/HBX)".

pore of the protein was reported as the highly probable binding site, with a spatial distribution of the most favorable sites in agreement with the most frequent salt bridge partners observed in all-atom MD simulations (14-3-3ζ Glu14, 14-3-3ζ Glu17, C-terminal c-Raf fragment His11).

In addition, our SAMC simulations make it possible to investigate the most probable binding region of HYD (Figure S3, first row) which was not done before.

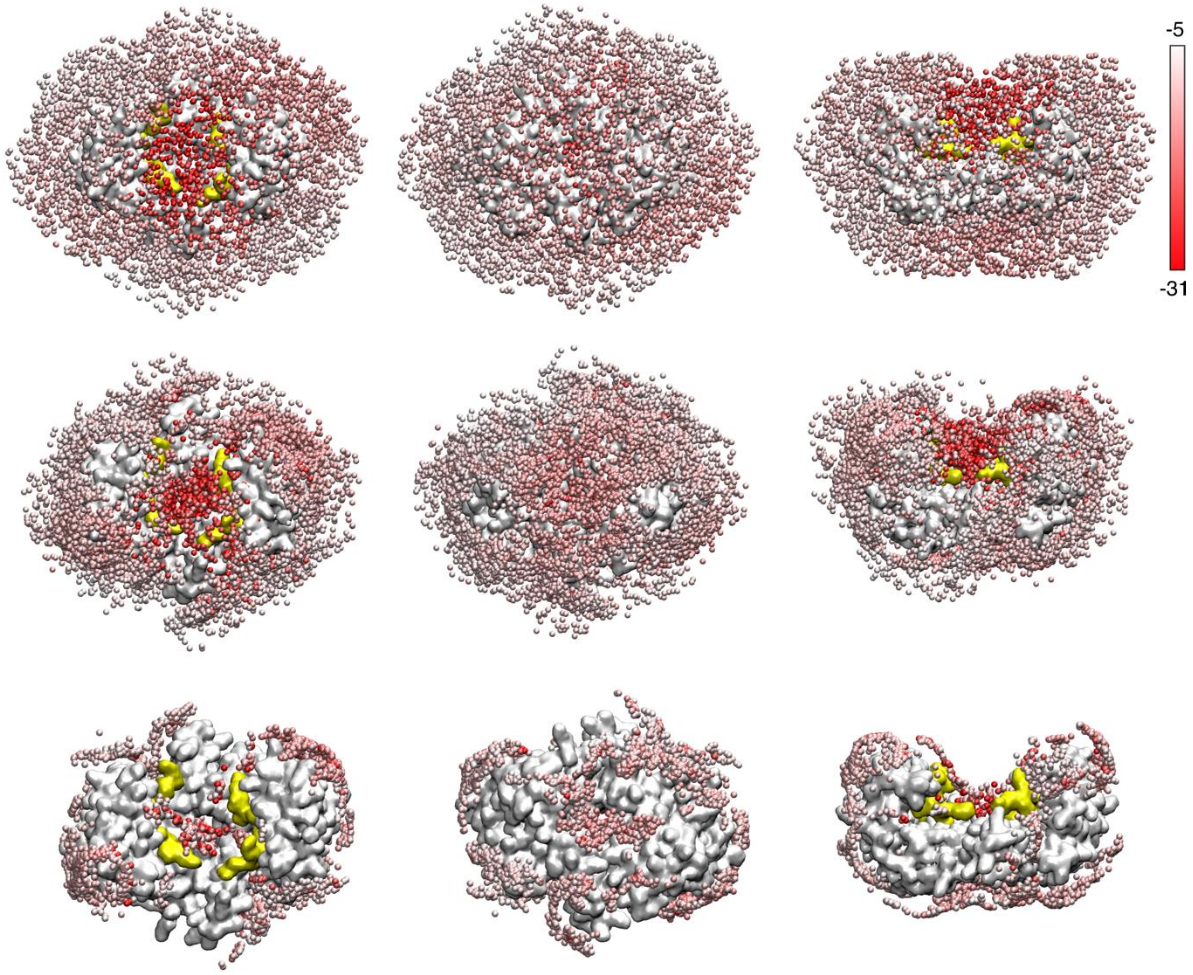

Figure S3: Sampled positions of the beads colored according to the total energy of the ligand from dark red (lowest energy) to white (highest energy). The values in the color bar are in units of kJ/mol. The three columns correspond to three different perspectives (top view in left column, bottom view in central column, side view in right column). Top row: HYD bead; middle row: GCP beads; bottom row: lysine beads.

Part 2: Applying SAMC approach to AIE/14-3-3ζ and AIE/14-3-3ζ/c-Raf complex

1. Electrostatic Potential Surface of 14-3-3ζ

The electrostatic potential surface of the protein in the presence and absence of C-Raf peptides is illustrated in Figure S4.

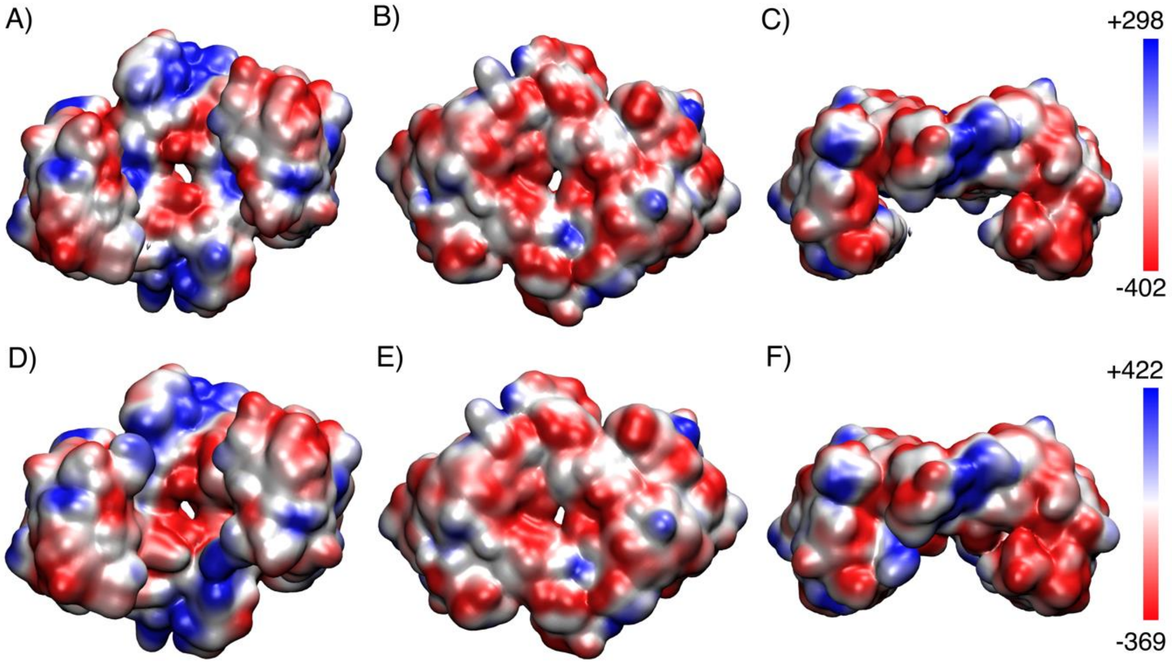

Figure S4: The electrostatic potential on the surface of 14-3-3ζ in the absence (A–C) and presence (D–F) of C-Raf peptides. The coloring method is based on the surface potential from dark red (most negative) to dark blue (most positive). The values in the color bar are in units of $k_BT/|e|$. The column in the left shows the top view, the one in the middle shows the bottom view, and the one in the right shows the side view.

2. Total energy of a simulation

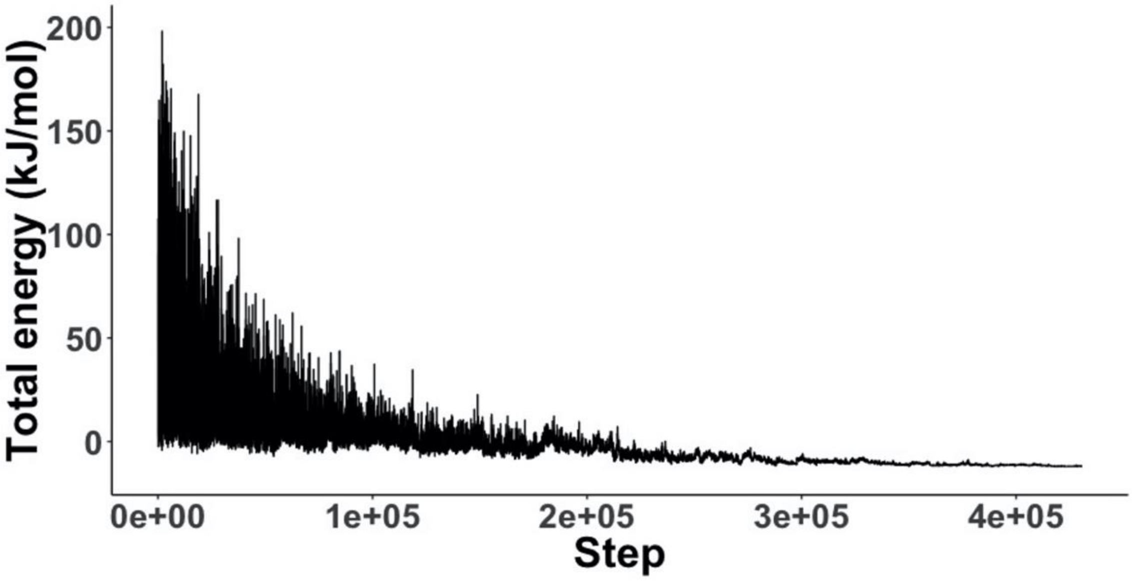

Figure S5: The total energy of the ligand throughout a representative single simulated annealing run.

3. Clustering

Applying a clustering method to the final positions of **1** provides a sanity check for our simulations. Considering the fact that 14-3-3ζ is a homodimer with C_2 symmetry, we expect for each binding site (or cluster of binding sites) that does not lie on the symmetry axis a symmetry related binding site (or cluster).

We use a robust silhouette-validated PAM (partitioning around medoids) clustering for this purpose [2]. Optimal number of clusters is extracted and visualized using the *fviz_nbclust* tool from R package *factoextra* version 1.0.7, and clustering is done using the *pam* tool from R package *cluster* version 2.1.0, in R version 3.5.3 [3].

The optimal number of clusters is obtained for two series of simulations (in the presence and absence of C-Raf peptides) using the Silhouette average method. The clustered data points obtained from each series of simulations are then mapped in 2D using their principal components as is illustrated in Figures S6 and S7.

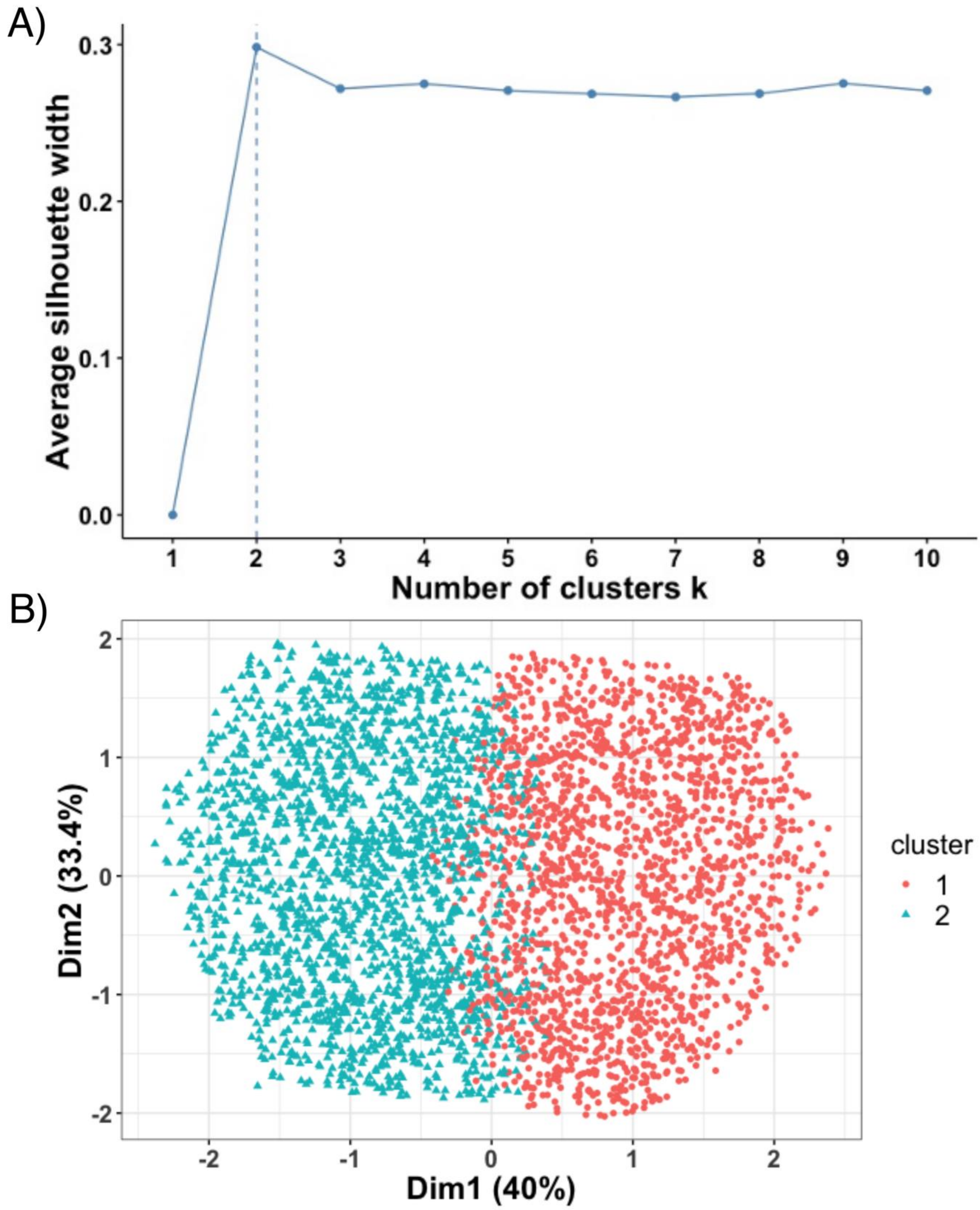

Figure S6: A) The final positions of AIE in the absence of C-Raf peptides can be optimally clustered in two groups as the Silhouette method suggests. B) A 2D projection of the clustered data on the two principal components.

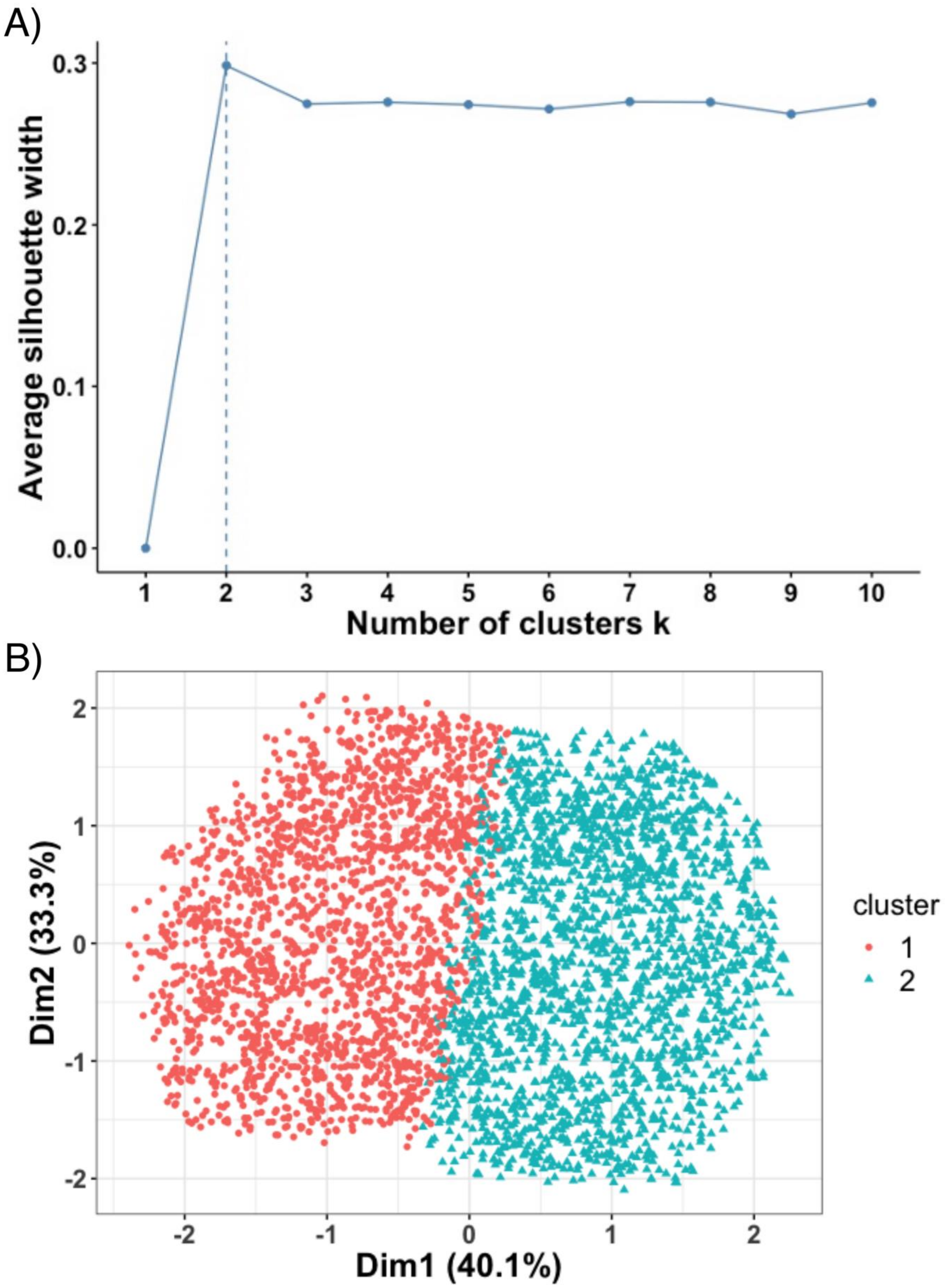

Figure S7: A) The final positions of AIE in the presence of C-Raf peptides can be optimally clustered in two groups as the Silhouette method suggests. B) A 2D projection of the clustered data on the two principal components.

4. General information and instrumentation

The used solvents for the synthesis and purification were dried and distilled before use. The purification of the water was performed with a TKA MicroPure ultrapure water system. The used chemicals for the synthesis were bought from commercial sources and used without any preparations (mainly from, Alfa Aesar, Fluorochem, Sigma-Aldrich, TCI). The reaction control was performed with TLC on normal phase silica gel plates (Macherey-Nagel, POLYGRAM SIL G/UV254) and UV light with a wavelength of 254 and 366 nm. The lyophilisation was performed with a Christ Alpha 1-4 LD plus freeze dryer. The determination of the Melting points was carried out with a Büchi Melting-Point B-540 apparatus and open-end glass capillary tubes. A Bruker DMX 300, DRX 500 and AVHD 600 spectrometer were used for the characterization of the final compounds. The measurements were performed at room temperature. The shifts of the signals are depending on the appropriate deuterated solvents. The chemical shifts are presented in parts per million. Mass spectrometry was performed on a Bruker BioTOF II or on a Thermo Fischer Orbitrap LTQ XL. HPLC-grade MeOH and ultrapure water were used as the mobile phase for the MPLC and HPLC. An Armen Instrument Spot Flash Liquid Chromatography MPLC apparatus was used for the purification and was equipped with a RediSep C_{18} reversed-phase column. The preparative HPLC consists of a TIDAS UV/NIR detector from J&M Analytic AG, P 2.1L pump from Knauer, a Labocol Vario-4000 collector and an Actus Triart C_{18} column from YMC (S-5 µm / 12 nm, 150 * 20 mm). The analytical HPLC for the determination of the purity contained a Dionex UltiMate 3000 pump, ASI100 Automated Sample Injector, UVD 340U detector and a YMC ODS-A column (15 cm × 3.0 mm, 5 µm). The IR spectra were recorded with an IR-Tracer-100 FTIR-Spectrophotometer from Shimadzu. The 14-3-3ζ protein was received from the working group of Dr. Christian Ottmann (Eindhoven University of Technology). The buffer contained 25 mM HEPES, 150 mM NaCl, 0.5 mM TCEP, 10 mM $MgCl_2$ and the pH value was 6.5.

Synthetic routes

Compound **A** [4] and the active ester **C** [5] were synthesized as described in the literature before. The activated azide **F** (tosylethylazide) can be prepared in analogy to a known two-step synthetic route [6]. The spectra obtained were in accordance with those reported in the literature.

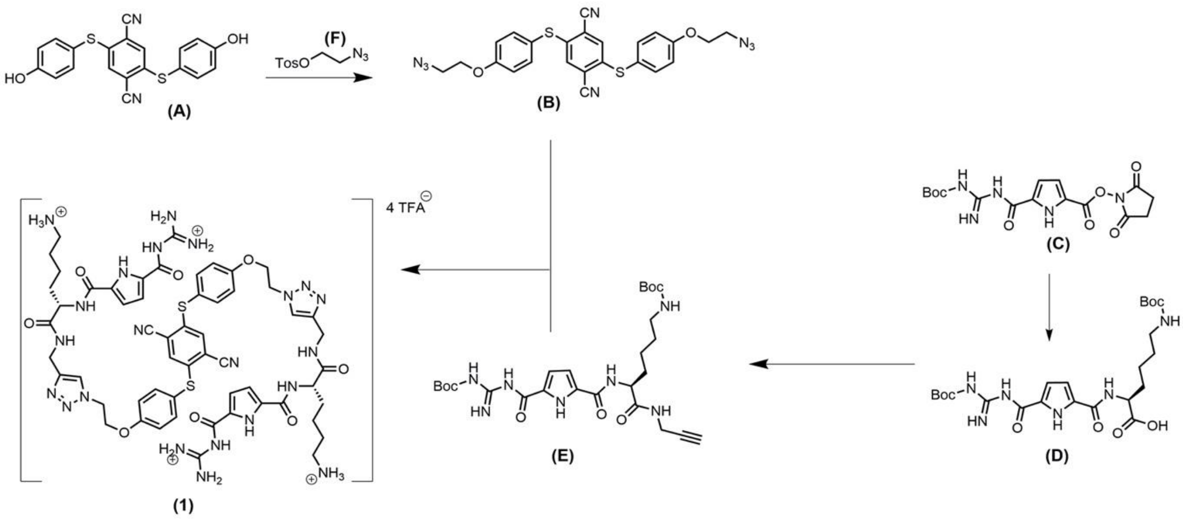

Figure S8: Synthetic routes to the corresponding desired GCP containing compound **1** featuring AIE- properties.

<u>Diazide (B)</u>

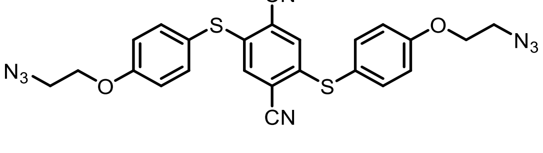

871 mg Compound **A** (1.0 equiv, 2.31 mmol,) was dissolved in 20 mL of DMF and 1.23 g tosylethylazide **F** (2.2 equiv, 5.10 mmol) was added followed by the addition of 1.93 g K_2CO_3 (6.1 equiv, 14 mmol). The mixture was stirred for 12 h at 50 °C followed by the addition of water. The precipitate was filtered and washed with water. The residue was recrystallized from boiling acetone yielding the desired product as a light yellow solid. Yield: 820 mg, 69%,

$M(C_{24}H_{18}N_8O_2S_2)$ = 514.58 g/mol, Mp: 166.3 °C, ^{1}H-NMR (400 MHz, Acetone-d_6) δ = 3.72 (t, J = 4.8 Hz, 4 H, CH_2), 4.32 (t, J = 4.7 Hz, 4 H, CH_2), 7.18 – 7.11 (m, 4 H, Ar-H), 7.33 (d, J = 0.9 Hz, 2 H, Ar-H), 7.62 – 7.57 (m, 4 H, Ar-H) ppm. ^{13}C-NMR (101 MHz, Acetone-d_6) δ = 50.8 (CH_2), 68.3 (CH_2), 115.8 (C_q), 116.1 (C_q), 117.4 (CH), 120.9 (C_q), 133.4 (CH), 137.7 (CH), 142.5 (C_q), 161.2 (C_q) ppm. IR (ATR): ν = 3082, 2938, 2876, 2224, 2100, 1591, 1572, 1491, 1462, 1449, 1408, 1395, 1346, 1302, 1285, 1242, 1221, 1175, 1148, 1101, 1053, 1007, 943, 912, 895, 851, 831, 812, 800, 723, 708, 673, 644. HR-MS (pos. ESI) m/z calculated $[M+Na]^{1+}$ 537.08863; found $[M+Na]^{1+}$ 537.08875.

BocGCPlys(Boc)OH (D)

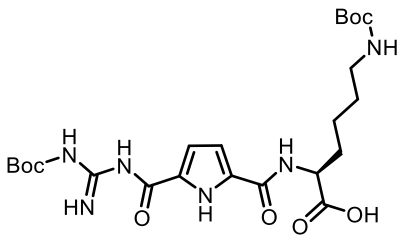

100 mg GCP-active-ester **C** (1.0 equiv, 0.25 mmol) and 62 mg H-lys(Boc)-OH (1.0 equiv, 0.25 mmol) were solved in 5 mL DMF. After that 0.35 mL Et_3N (10 eq, 2.52 mmol, 254 mg) were added at 0 °C and stirred at room temperature for 24 h. 100 mL ethyl acetate was added to the reaction mixture and the organic phase was washed three times each with 50 mL 0.5 M $NaHSO_4$ solution, 50 mL brine, and 50 mL water. The organic phase was dried with $MgSO_4$, filtered and the solvent removed with the evaporator. The residue was freeze dried. The product was obtained as a white solid. Yield: 115 mg, 87%, $M(C_{23}H_{36}N_6O_8)$ = 524.58 g/mol. R_f = 0.15 (1Cy/ EA/ 0.1% AcOH), Mp: 270 °C (decomposition), ^{1}H-NMR (300 MHz, DMSO-d_6): δ = 1.28-1.56 (m, 4H, lys-γ, δCH_2), 1.34 (s, 9H, Boc), 1.46 (s, 9H, Boc), 1.77 (m, 2H, lys-βCH_2), 2.88 (m, 2H, lys-εCH_2), 4.33 (m, 1H, lys-αCH), 6.76 (m, 1H, amide N-H), 6.84 (s, 1H, pyrrole-H), 6.89 (s, 1H, pyrrole-H), 8.49 (d, J = 7.9 Hz, H, amide-N-H) 8.72 (s, 1H, N-H), 9.41 (s, 1H, N-H), 11.69 (s, 3H, 2N-H + COO-H) ppm, ^{13}C-NMR (150 MHz, DMSO-d_6): δ = 23.0 (lys-

γCH_2), 27.7 (Boc-CH_3), 28.2 (Boc-CH_3), 29.1 (lys-δCH_2), 30.6 (lys-βCH_2), 40.1 (lys-εCH_2), 52.0 (lys-αCH), 77.3 (Boc-C-CH_3), 112.8 (pyrrole), 113.7 (pyrrole),155.5 (C_q), 159.5 (C_q), 173.6 (C_q) ppm, IR max/cm^{-1}: 3323 (b), 2976 (w), 2934 (w), 1688 (s), 1634 (w), 1553 (w), 1472 (w), 1393 (w), 1368 (w), 1242 (s), 1142 (s), 1047 (w), 993 (w), 833 (w), 754 (w). HR-MS (pos. ESI) m/z calculated $[M+H]^{1+}$ 525.2667, $[M+Na]^{1+}$ 547.2487; found $[M+H]^{1+}$ 525.2682, $[M+Na]^{1+}$ 547.2495.

BocGCPlys(Boc)alkyne (E)

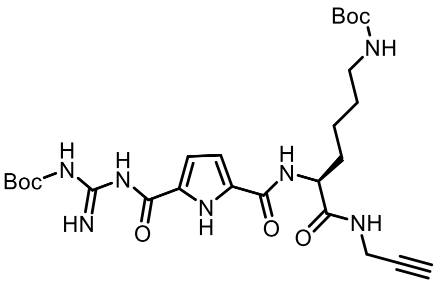

100 mg BocGCPlys(Boc)OH **D** (1.0 equiv, 0.19 mmol), 0.13 mL Et_3N (5.0 equiv, 0.95 mmol, 96.5 mg) and 86 mg HBTU (1.2 equiv, 0.23 mmol) were dissolved in 10 mL DMF and stirred for 30 min. After that, 14.5 μL propargylamine (1.2 equiv, 0.23 mmol, 12 mg) was added and the reaction mixture was stirred overnight. 100 mL ethyl acetate was added and the organic phase was washed three times each with 50 mL 0.5 M $NaHSO_4$-solution, 50 mL brine, and 50 mL water. The organic phase was dried with $MgSO_4$, filtered and the solvent removed with the evaporator. The crude product was purified with normal phase chromatography (1Cy/ 1EA). The product was freeze dried and was obtained as a white solid. Yield: 80 mg, 74%, M($C_{26}H_{39}N_7O_7$) = 561.64 g/mol, Rf = 0.40 (1Cy/ 1EA), Mp: 190 °C (decomposition). ^{1}H-NMR (400 MHz, DMSO-d_6): = 1.30-1.55 (m, 4H, lys-γ, δCH_2), 1.35 (s, 9H, Boc), 1.46 (s, 9H, Boc), 1.67 (m, 2H, lys-βCH_2), 2.88 (m, 2H, lys-εCH_2), 3.09 (m, 1H, alkyne-H), 3.85 (m, 2H, alkyne), 4.37 (m, 1H, lys-αCH), 6.76-6.82 (s+m, 3H, 2 pyrrole-H + amide N-H), 8.46 (m, 2H, amide

N-H), 8.57 (s, 1H, N-H) 9.33 (s, 1H, N-H), 10.91 (s, H, N-H), 11.53 (s, 1H, N-H) ppm. ^{13}C-NMR (101 MHz, DMSO-d_6): δ = 23.0 (lys-γCH_2), 27.8 (Boc-CH_3), 27.9 (alkyne CH_2), 28.2 (Boc-CH_3), 29.2 (lys-δCH_2), 31.6 (lys-βCH_2), 40.1 (lys-εCH_2), 52.6 (lys-αCH), 72.9 (alkyne CH), 77.3 (Boc-C-CH_3), 81.1 (alkyne C_q), 112.9 (pyrrole), 155.5 (C_q), 159.4 (C_q), 171.6 (C_q) ppm. IR max/cm^{-1}: 3370 (b), 3285 (b), 2976 (w), 2930 (w), 2866 (w), 2349 (w), 1634 (s), 1532 (s), 1456 (w), 1393 (w), 1368 (w), 1289 (s), 1240 (s), 1148 (s), 1047 (w), 841 (s), 756 (w). HR-MS (pos. ESI) m/z calculated $[M+H]^{1+}$ 562.2984, $[M+Na]^{1+}$ 584.2803; found $[M+H]^{1+}$ 562.2965, $[M+Na]^{1+}$ 584.2779.

<u>(GCPlys)$_2$AIE (1)</u>

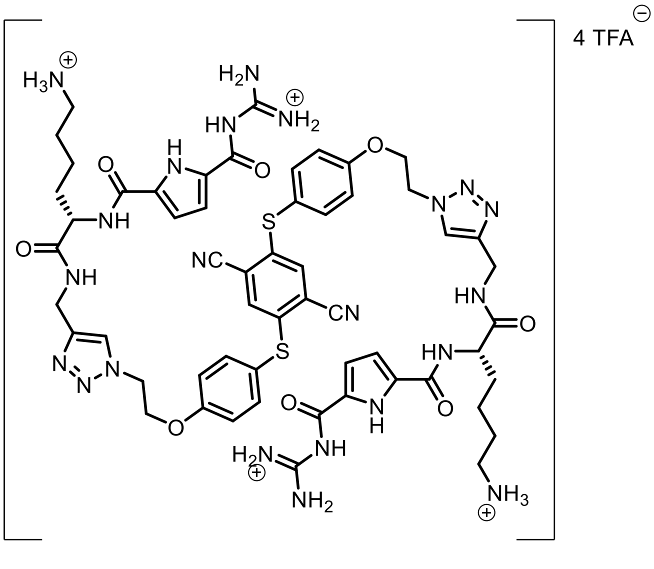

70 mg BocGCPlys(Boc)alkyne **E** (2.0 equiv, 0.12 mmol) and 32 mg AIE-diazide **B** (1.0 equiv, 0.06 mmol) were dissolved in 40 mL distilled THF under argon atmosphere. Then, 10 mL degassed water was added. A solution of 3.7 mg copper sulfate pentahydrate (0.1 equiv, 6.23 μmol) and 3.0 mg sodium ascorbate (0.1 equiv, 6.23 μmol) in 1 mL degassed water were added. Every hour 3.0 mg sodium ascorbate was added until the reaction started (TLC). After the complete conversion of the starting materials, the solvent was removed with the evaporator. The residue was dissolved in 10 mL DCM and 20 mL TFA was added. The reaction mixture

was stirred overnight and the solvent was removed with the evaporator. The residue was freeze dried. Finally, the crude product was purified with reversed-phase chromatography (MPLC RP_{18}: gradient from 30% MeOH/70% H_2O/0.1% TFA to 100 % MeOH/0.1% TFA; HPLC RP_{18}: gradient from 40% MeOH/60% H_2O/0.1% TFA to 60% MeOH/40% H_2O/0.1% TFA. The product was obtained as a yellow solid as the corresponding TFA salt (4 TFA^-). Yield: 9 mg, 9%, M($C_{56}H_{64}N_{22}O_8S_2$) = 1237.39 g/mol. Purity: 96-97 %, Mp: 230 °C (decomposition), ^{1}H-NMR (600 MHz, DMSO-d_6, trifluoroacetate-salt): δ = 1.33-1.38 (br. m, 4H, lys-γCH_2), 1.54 (m, 4H, lys-δCH_2), 1.65 (m, 2H, lys-βCH_2), 1.75 (m, 2H, lys-βCH_2), 2.76 (m, 4H, lys-εCH_2), 4.33 (m, 4H, triazole-$\underline{CH_2}$-N), 4.44 (m, 6H, triazole-$\underline{CH_2}$-CH_2, αC-H), 4.76 (m, 4H, O-$\underline{CH_2}$-CH_2), 6.63 (s, 2H, N-H), 6.89 (s, 2H, pyrrole-H), 7.06 (d, J = 8.3 Hz, 4H, Ar-H), 7.20 (s, 2H, pyrrole-H), 7.35 (s, 2H, Ar-H), 7.48 (d, J = 8.3 Hz, 4H, Ar-H), 7.79 (br. s, 6H, lys-NH_3^+), 7.98 (s, 2H, triazole-H), 8.59 (d, J = 7.0 Hz, 2H, NH) , 8.63 (s, 2H, NH), 8.80 (br. s, 6H, guanidine-NH^+), 11.64 (s, 2H, guanidine amide-NH), 12.49 (s, 2H, pyrrole-NH) ppm. ^{13}C-NMR (150 MHz, DMSO-d_6, trifluoroacetate-salt): δ = 22.6 (lys-γCH_2), 26.6 (lys-δCH_2), 31.4 (lys-βCH_2), 34.3 (triazole-$\underline{CH_2}$-N), 38.7 (lys-εCH_2), 48.9 (O-$\underline{CH_2}$-CH_2), 52.7 (lys-αCH), 66.5 (triazole-$\underline{CH_2}$-CH_2), 113.6 (pyrrole-CH), 114.2 (TFA^-), 115.0 (pyrrole-CH), 115.2, 115.2 (C_q), 116.2 (TFA^-), 116.6 (Ar-CH), 118.3 (TFA^-), 119.8, (C_q), 120.2 (TFA^-) 123.4 (triazole-CH), 125.7, 132.3 (C_q), 132.9 (Ar-CH), 136.2 (Ar-CH), 140.9 (C_q), 144.9 (C_q), 155.4 (C_q), 158.2 (TFA^-), 158.4 (TFA^-), 158.7 (TFA^-) 159.0 ($C_{q,}$, overlap with TFA^-), 159.3 (C_q), 171.5 (C_q) ppm. IR max/cm^{-1}: 3310 (b), 3080 (b), 2945 (b), 2104 (w), 1655 (s), 1593 (w), 1493 (w), 1474 (w), 1452 (w), 1431 (w), 1344 (w), 1246 (w), 1175 (s), 1128 (s), 1049 (w), 1007 (w), 907 (w), 831 (w), 799 (w), 752 (w), 719 (s), 704 (w). HR-MS (pos. ESI) m/z calculated $[M+2H]^{2+}$ 619.2432, $[M+3H]^{3+}$ 413.3036, $[M+4H]^{4+}$ 310.1252; found $[M+2H]^{2+}$ 619.2434, $[M+3H]^{3+}$ 413.1643, $[M+4H]^{4+}$ 310.1247.

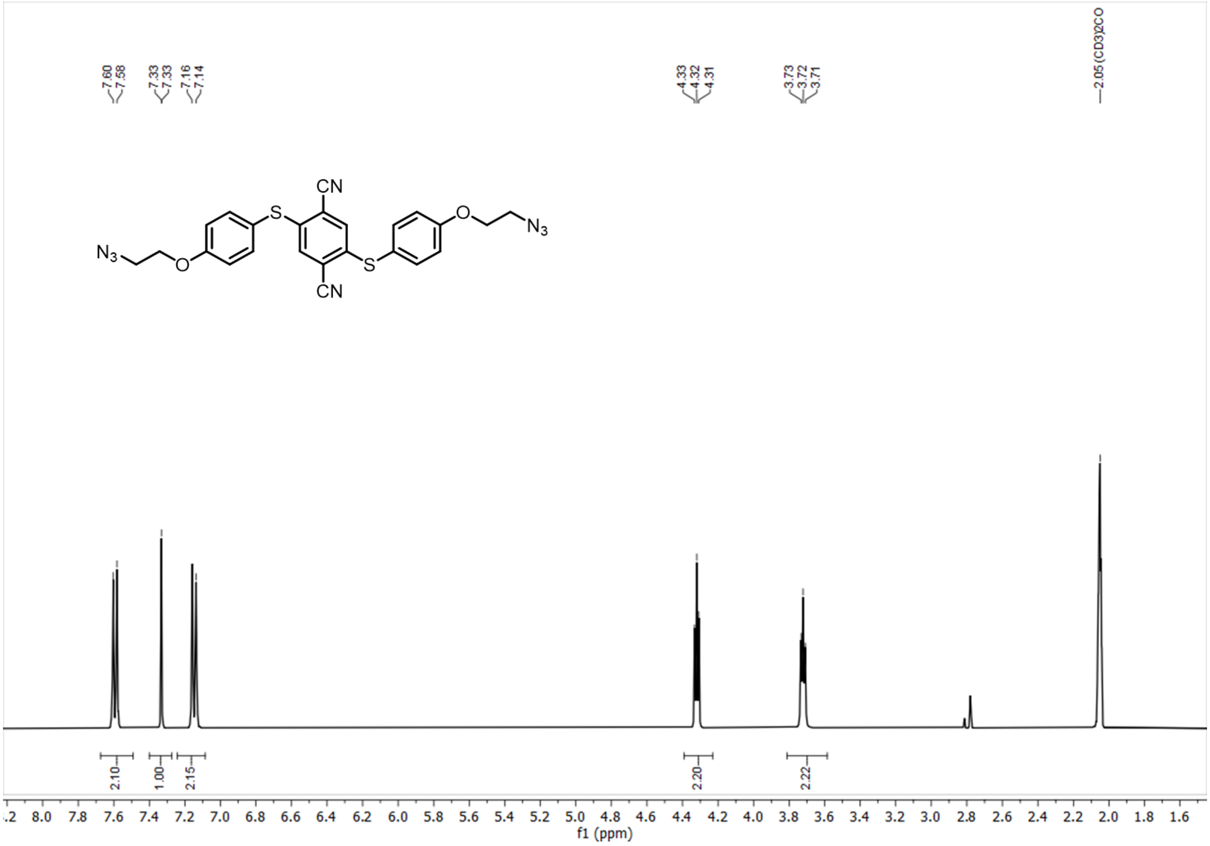

Figure S9: ^{1}H-NMR spectrum of diazide **B** (400 MHz, acetone-d_6).

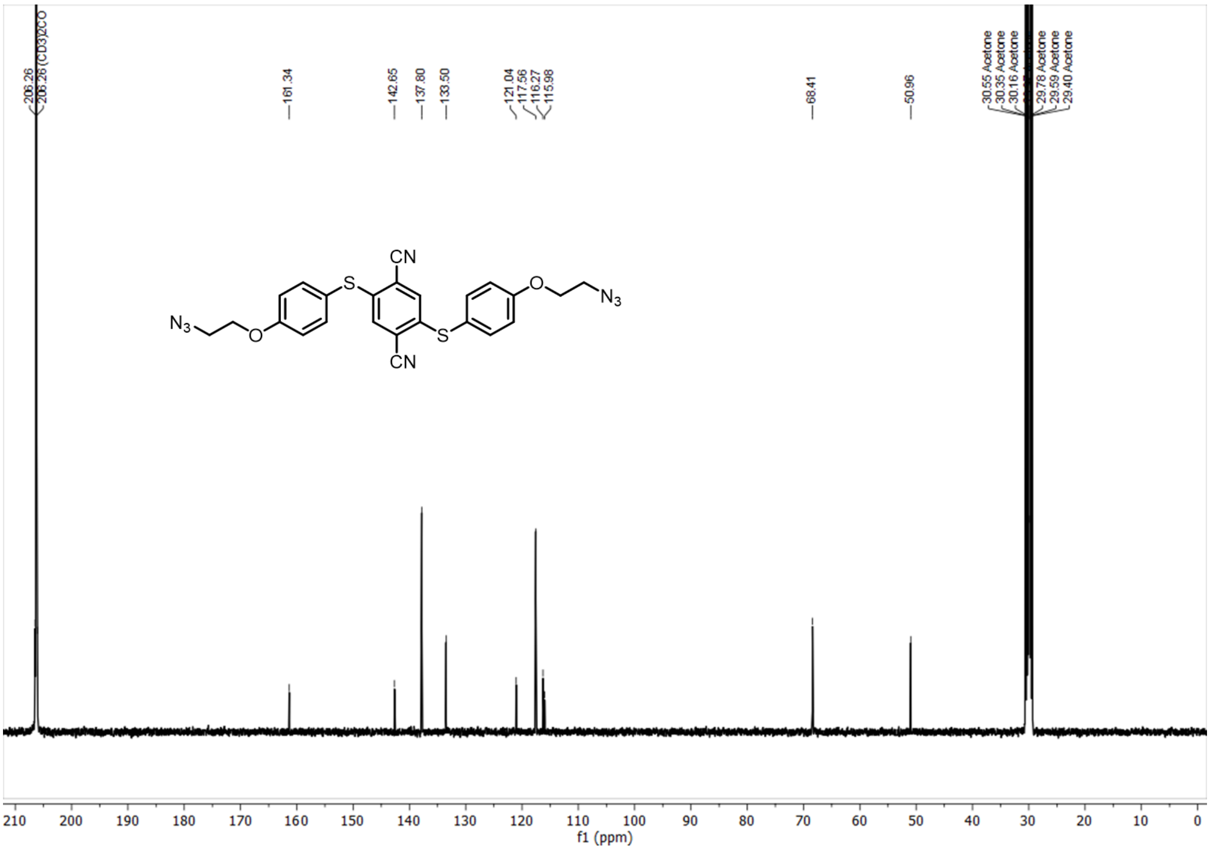

Figure S10: ^{13}C-NMR spectrum of diazide **B** (101 MHz, acetone-d_6).

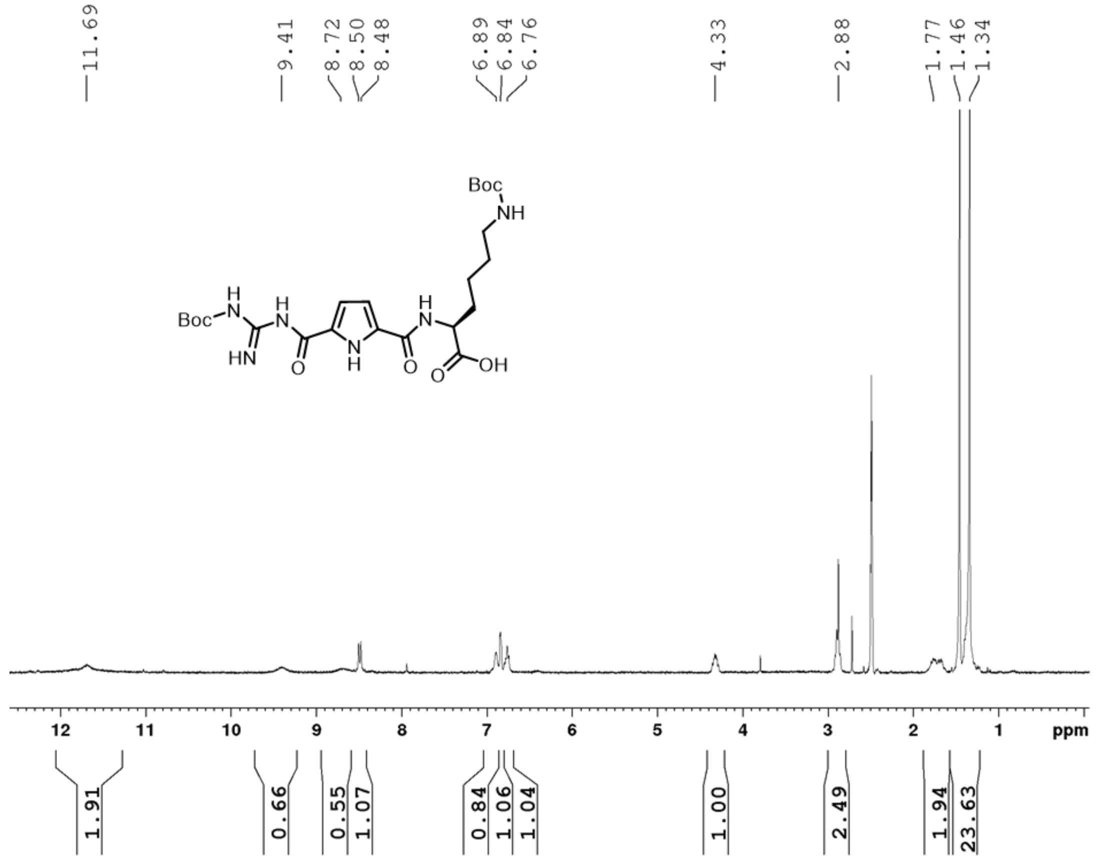

Figure S11: ^{1}H-NMR spectrum of **D** (300 MHz, DMSO-d_6).

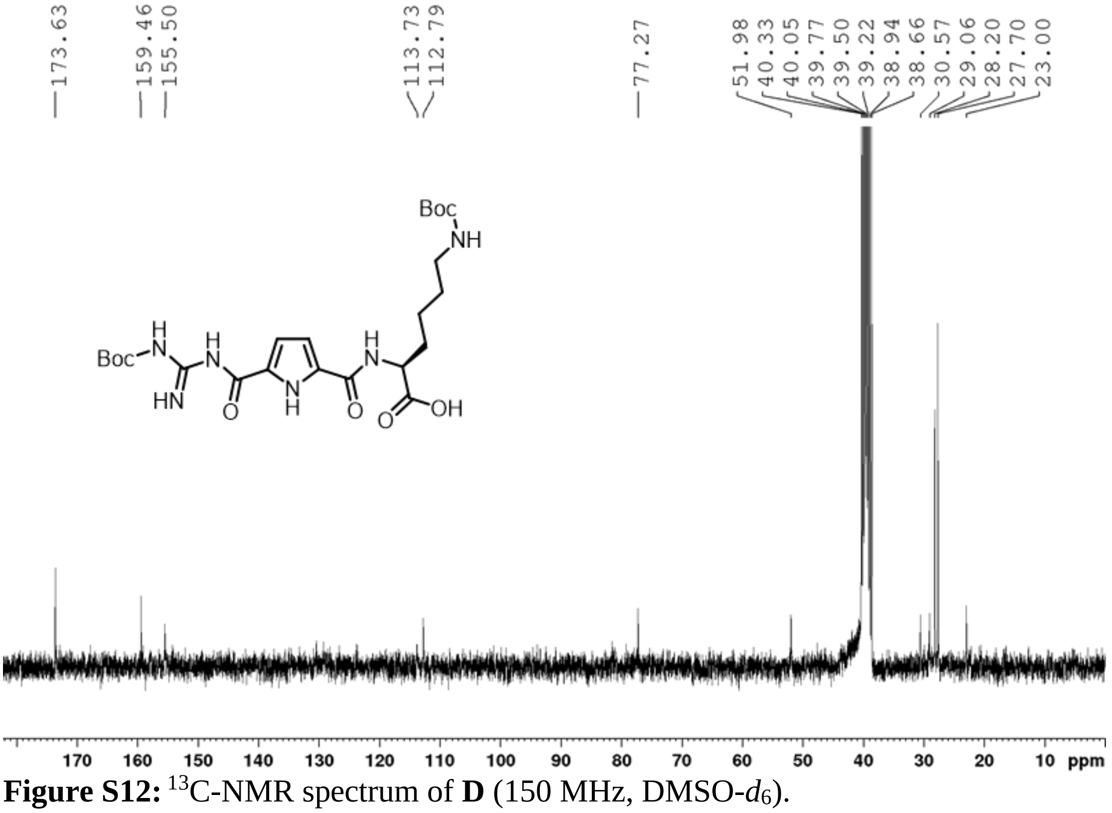

Figure S12: ^{13}C-NMR spectrum of **D** (150 MHz, DMSO-d_6).

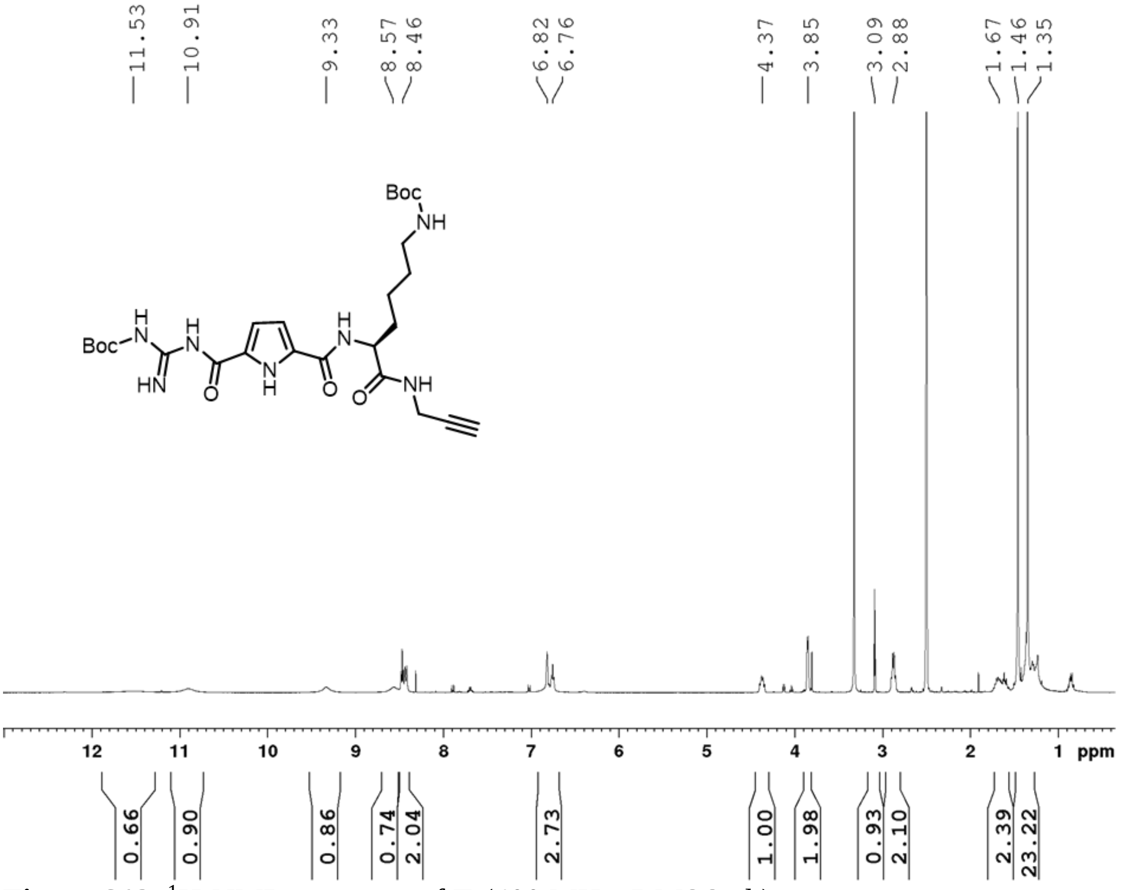

Figure S13: ^{1}H-NMR spectrum of **E** (400 MHz, DMSO-d_6).

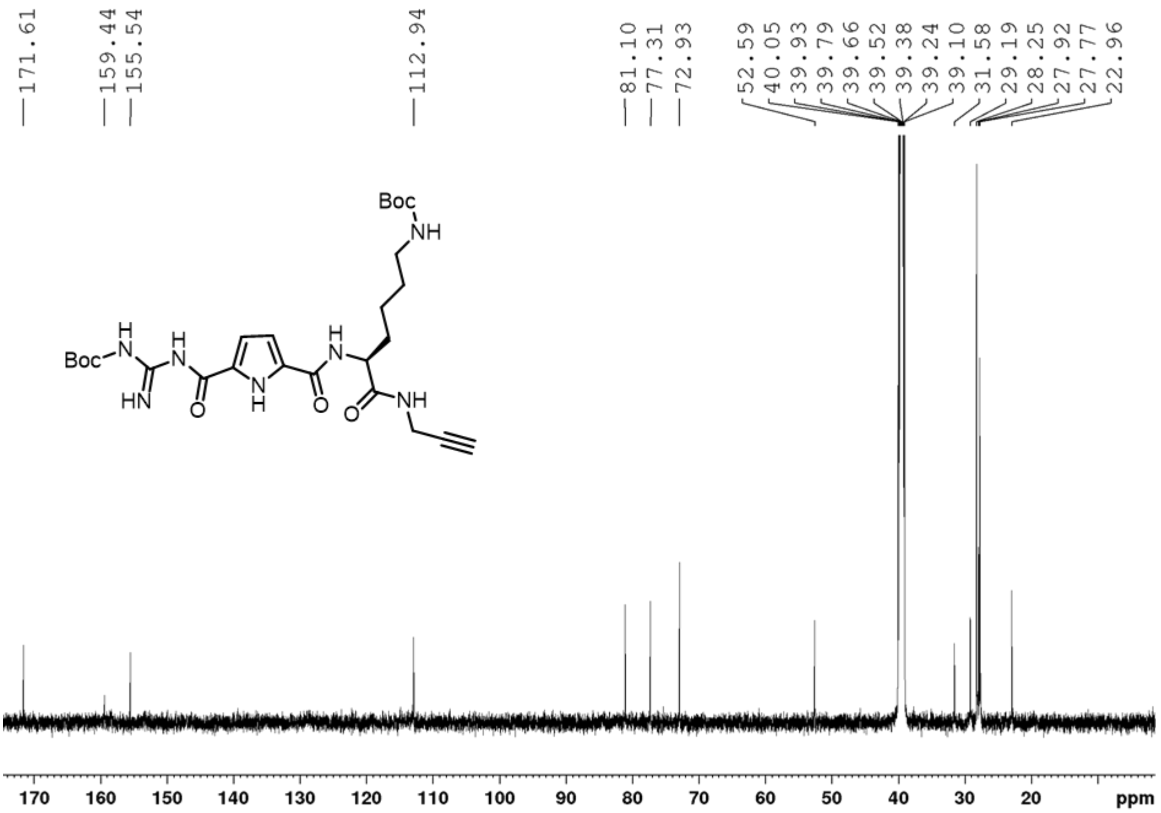

Figure S14: ^{13}C-NMR spectrum of **E** (101 MHz, DMSO-d_6).

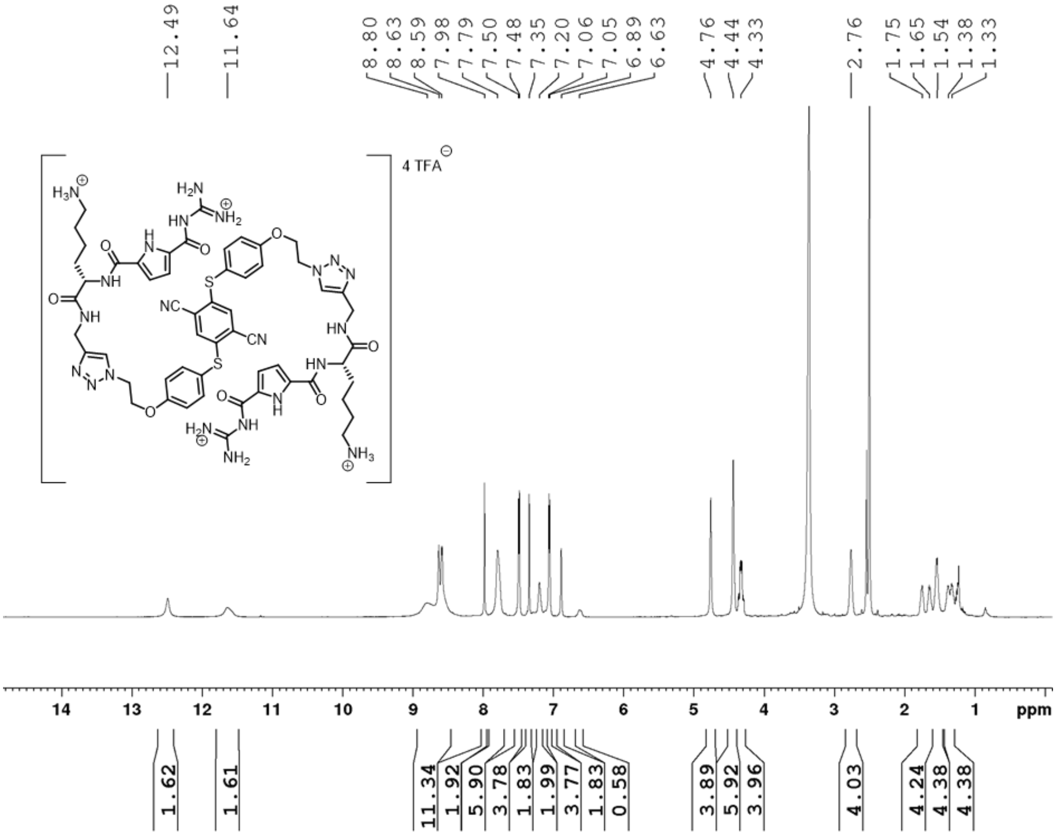

Figure S15: ^{1}H-NMR spectrum of **1** (600 MHz, DMSO-d_6 as TFA salt). 2.54 ppm: DMSO [7], 1.2 and 0.8 ppm: H-grease [8].

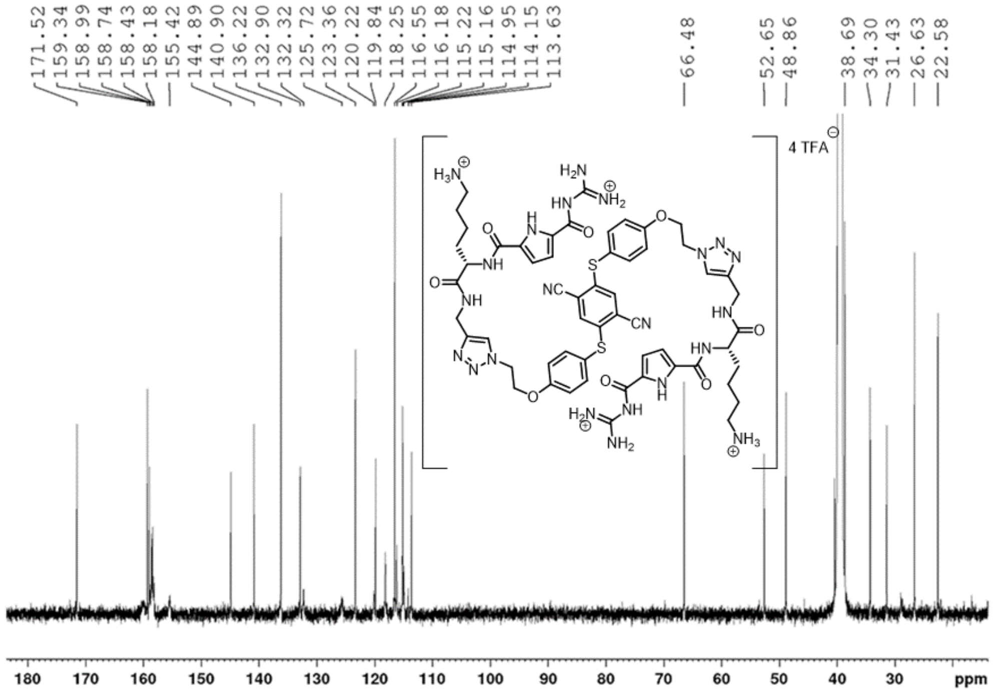

Figure S16: ^{13}C-NMR spectrum of **1** (150 MHz, DMSO-d_6 as TFA salt).

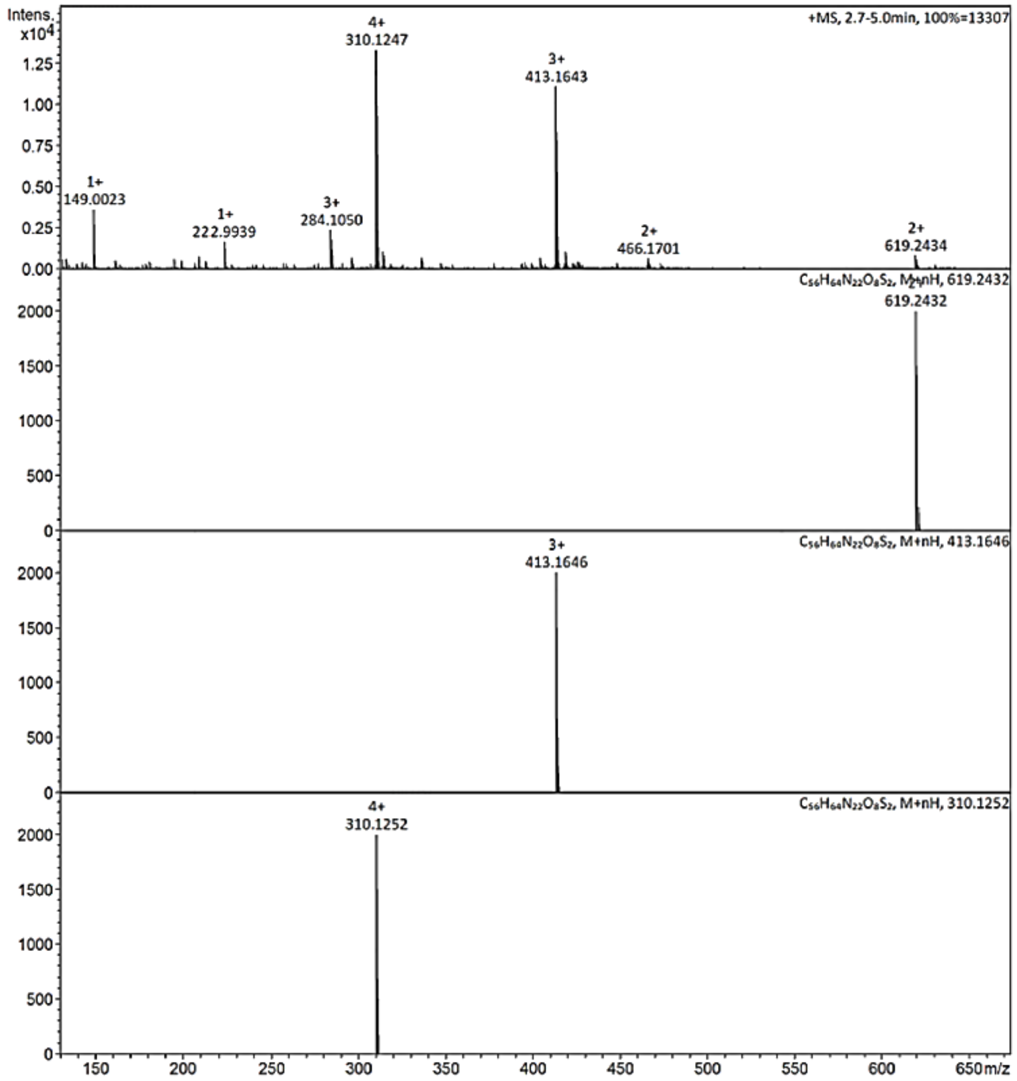

Figure S17: HR-ESI mass spectrum of **1** (positive ion mode, MeOH) and predicted mass spectra of the corresponding peaks.

Sample Name:			Quant. Method:	HPLC_1\Thies\MaKi180\Maki 180prep.SEQ			
Vial Number:	RB6	Value A	0	%	Wasser (BisTris) pH 6.1	Injection Volume:	10.0
Recording Time:	14/06/2021 16:30	Value B	0	%		Channel:	UV_VIS_4
Run Time (min):	37.00	Value C	60	%		Wavelength:	290
Flow (ml/min):	0.430	Value D	40	%		Bandwidth:	1

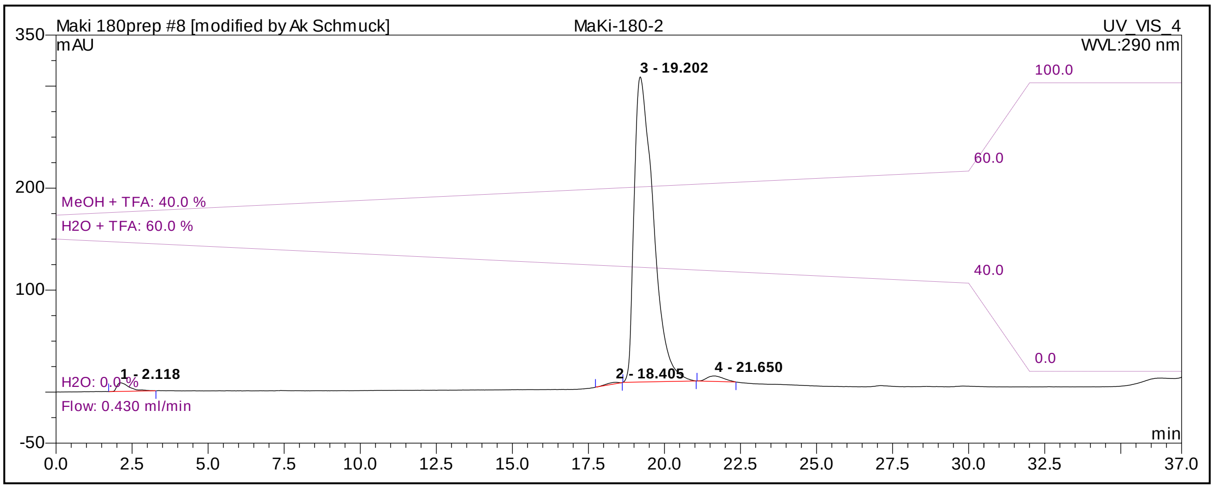

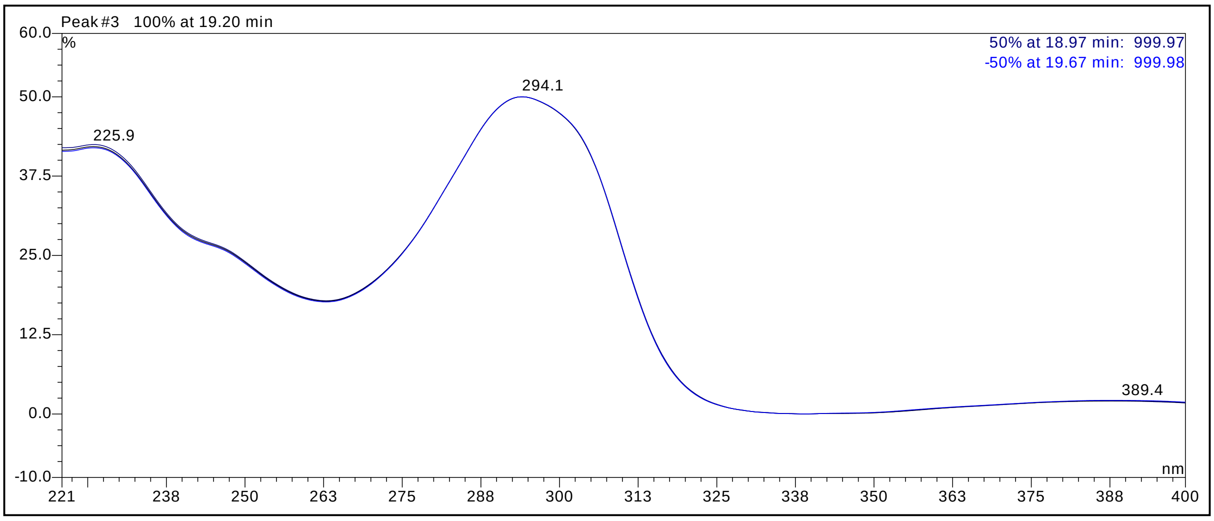

No.	Ret.Time	eak Nam	Height	Area	Rel.Area	Rel.Height
	min		mAU	mAU*min	%	%
1	2.12	n.a.	8.398	4.023	1.77	2.67
2	18.40	n.a.	1.369	0.739	0.33	0.44
3	19.20	n.a.	299.110	219.098	96.41	95.19
4	21.65	n.a.	5.335	3.400	1.50	1.70
Total:			*314.2110*	*227.26*	*100.00*	*100.00*

Figure S18: HPLC analysis of **1** gradient from 40% MeOH/ 60% H_2O/TFA (0.1%) to 60% MeOH/ 40% H_2O/TFA (0.1%) in 30 min, flow rate: 0.43 mL/min, retention time t_R = 19.202 min, purity: > 96% (peak integration based on UV-detection at 290 nm).

Sample Name:			Quant. Method:	HPLC_1\Thies\MaKi180\Maki 180prep.SEQ		
Vial Number:	RB6	Value A	0 %	Wasser (BisTris) pH 6.1	Injection Volume:	10.0
Recording Time:	14/06/2021 16:30	Value B	0 %		Channel:	UV_VIS_2
Run Time (min):	37.00	Value C	60 %		Wavelength:	254
Flow (ml/min):	0.430	Value D	40 %		Bandwidth:	1

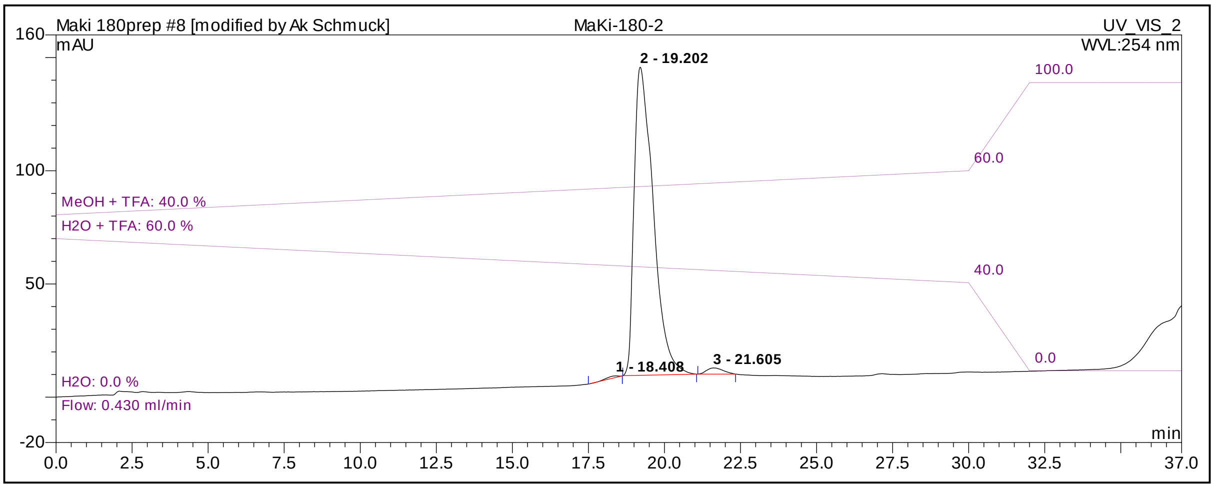

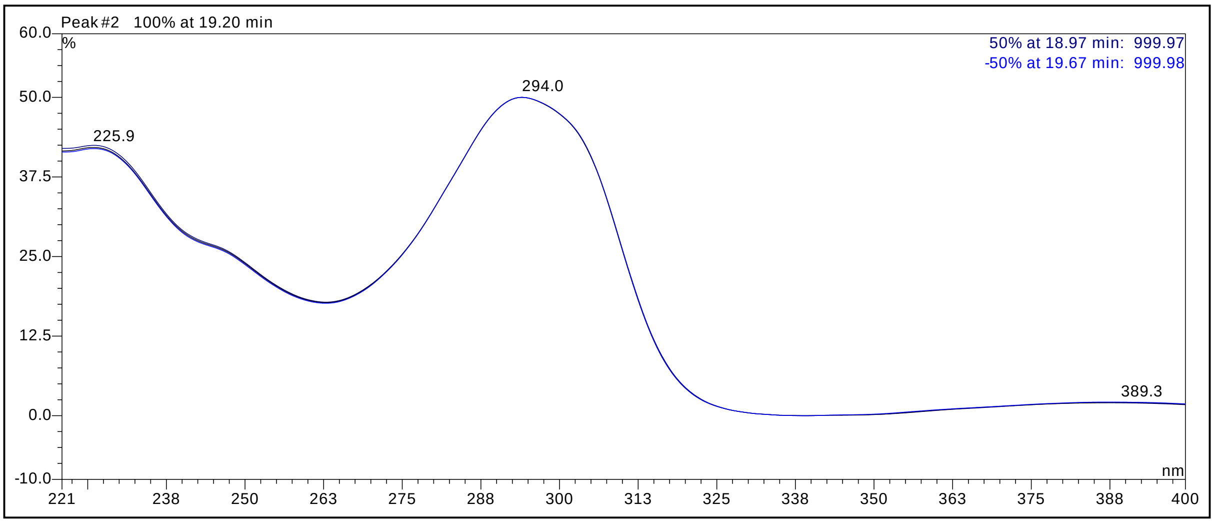

No.	Ret.Time	Peak Name	Height		Area	Rel.Area	Rel.Height
	min		mAU		mAU*min	%	%
1	18.41	n.a.	0.699		0.369	0.36	0.50
2	19.20	n.a.	136.084		99.467	97.94	97.54
3	21.60	n.a.	2.733		1.728	1.70	1.96
Total:			*139.5156*		*101.56*	*100.00*	*100.00*

Figure S19: HPLC analysis of **1** gradient from 40% MeOH/ 60% H_2O/TFA (0.1%) to 60% MeOH/ 40% H_2O/TFA (0.1%) in 30 min, flow rate: 0.43 mL/min, retention time t_R = 19.202 min, purity: > 97% (peak integration based on UV-detection at 254 nm).

5. Native gel electrophoresis

For native gel electrophoresis, a discontinuous polyacrylamide gel was prepared (1.5 mm, 10 wells) using 12.5% (w/v) acrylamide for the separation gel and 4% (w/v) acrylamide for the stacking gel (Table S1). The experimental setup (Table S2) included a 1 mM stock solution of 14-3-3ζ in native running buffer (Table S1) and 1 mM of ligand **1** dissolved in water. To avoid alteration in the protein structure caused by dilution with the ligand, we used 4x concentrated sample buffer to equalize the buffer conditions and added the protein last (Table S1). The samples were centrifuged at 10000 ×G for 1 Min (ThermoFisher) to spin down potential aggregates before they were loaded onto the gel. Negative controls contained either protein (well #3) or ligand (well #4) to assure that no aggregates are present in the respective stock solutions and that the localization shifts of both colorimetric protein and fluorescent ligand bands are a result of the interaction. Electrophoresis was performed with a Mini-Protean Tetra Cell (BioRad) for 2 hours at constant 175 V. To avoid protein denaturation caused by the development of heat during electrophoresis, the buffer was constantly cooled and stirred. The fluorescence of the ligand was detected using a ChemiDoc (BioRad) or E-Box VX2 (Vilber) system. To additionally allow visualization of the protein bands, we subsequently performed a coomassie staining of the gel. The gel was incubated in the staining solution for 1 hour and destained overnight. The composition of the gels, buffers, and solutions are summed up in the following tables (Table S1 and S2).

Table S1: Composition of the gel, buffers and solutions.

**Component**	**Composition**
Native stacking gel buffer(4**)	0.5 M tris-HCl, pH 6.8
Native separation gel buffer (4**)	1.5 M tris-HCl, pH 8.8
Native running buffer (10*)	25 mM tris-HCl, 192 mM glycine, pH 8.8
Native sample buffer	2.5 mM tris-HCl, 19.2 mM glycine, 30% (v/v) glycerol, pH 8.8
Coomassie staining solution	10% (v/v) acetic acid, 40% (v/v) ethanol, 0.1% (w/v) coomassie G-250
Destaining solution	10% (v/v) acetic acid, 40% (v/v) ethanol
4% stacking gel	2.5 mL ddH2O, 1.3 mL native stacking gel buffer, 0.65 mL 30% acrylamide, 5 µL TEMED, 50 µL 10% APS
12.5% separation gel	1.6 mL ddH2O, 1.3 mL native stacking gel buffer, 2.1 mL 30% acrylamide, 5 µL TEMED, 50 µL 10% APS

Table S2: Experimental setup.

**Well**	**#2 (empty)**	**#3**	**#4**	**#5 (empty)**	**#6**	**#7 (empty)**	**#8**	**#9 (empty)**	**#10**
Sample buffer (4*)		3 µL	3 µL		3 µL		3 µL		3 µL
Ligand **1**		/	10 µL		1 µL		5 µL		10 µL
Running buffer		10 µL	/		9 µL		5 µL		/
Protein 14-3-3ζ		1 µL	/		1 µL		1 µL		1 µL

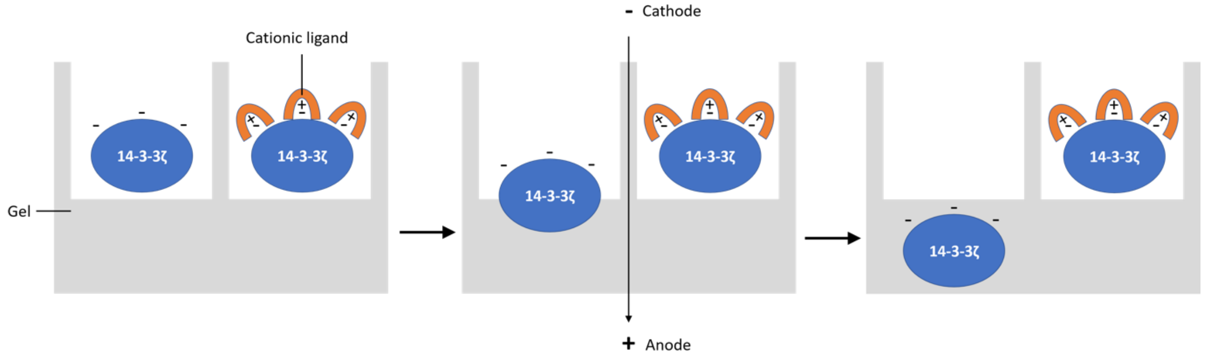

Figure S20: Schematic overview of the effects of ligands binding on the protein's migratory behavior during polyacrylamide gel electrophoresis. The cationic ligand is suggested to mask the negative charges in the protein and thereby prevents its movement through the gel towards the anode.

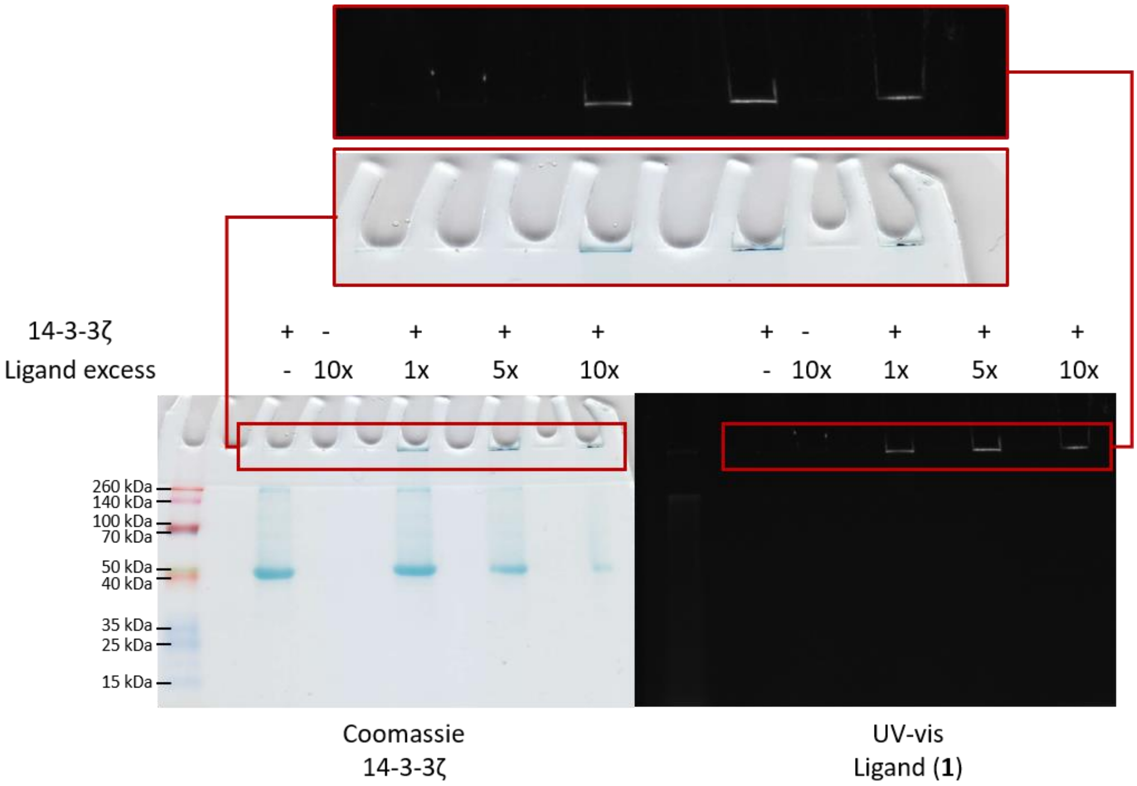

Figure S21: Binding of ligand **1** prevents the protein 14-3-3ζ from entering the gel. The amount of protein (blue bands) that migrated into the gel decreases with the amount of ligand added. At the same time, the ligand (UV) was only detectable in the well if protein was added. In addition, the ligand in the well co-localized with the protein retained in the well. Of note, not the complete amount of protein present in the well was detectable.

6. Fluorescence / UV–vis titration and Job plot analysis

In all spectroscopic measurements, the following buffer was used to ensure correct folding and stability of the 14-3-3ζ protein: 25 mM HEPES, 150 mM NaCl, 10 mM $MgCl_2$, 0.5 mM TCEP, pH 6.5.

The fluorescence spectroscopy was performed in quartz cuvettes using a Shimadzu RF-6000 spectro fluorophotometer and UV–vis measurements were carried out on a Cary 300 Bio UV-Visible Spectrophotometer at room temperature. The excitation was monitored at 390 nm. The titration started with 5 μM ligand in the cuvette and stepwise protein was added (up to 8 equiv), leading to an emission increase. Visible precipitation occurred at 7 equiv of 14-3-3ζ and the

fluorescence signal was not constant anymore. The emission maximum was found to be at ≈470 nm (blue emission). To avoid dilution of ligand **1** during the titration the protein stock solution contained 5 µM of ligand **1**.

Concentration and time dependent measurements of **1**, using UV–vis and fluorescence spectroscopy, showed a linear behavior in the concentration ranged used for the fluorescence titration and Job plot analysis. As consequence we assume no intra- or intermolecular stacking of the aromatic moieties in the concentration range used.

The analysis was performed in OriginPro 2019b with the quadratic equation:

$$A_0 + \frac{A\left((L + x + K_d) - \sqrt{(L + x + K_d)^2 - 4xL}\right)}{2L}$$

with L= ligand concentration, x= protein concentration, K_d= dissociation constant, A_0= starting signal and A= amplitude [9, 10].

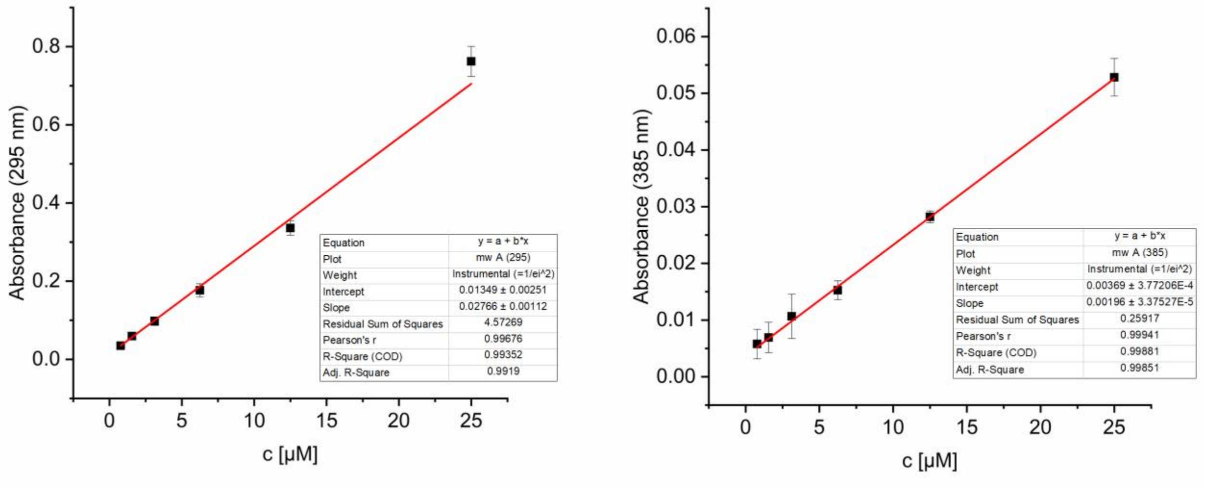

Figure S22: Determination of molar extinction coefficient at 295 and 385 nm for ligand **1** in buffer solution.

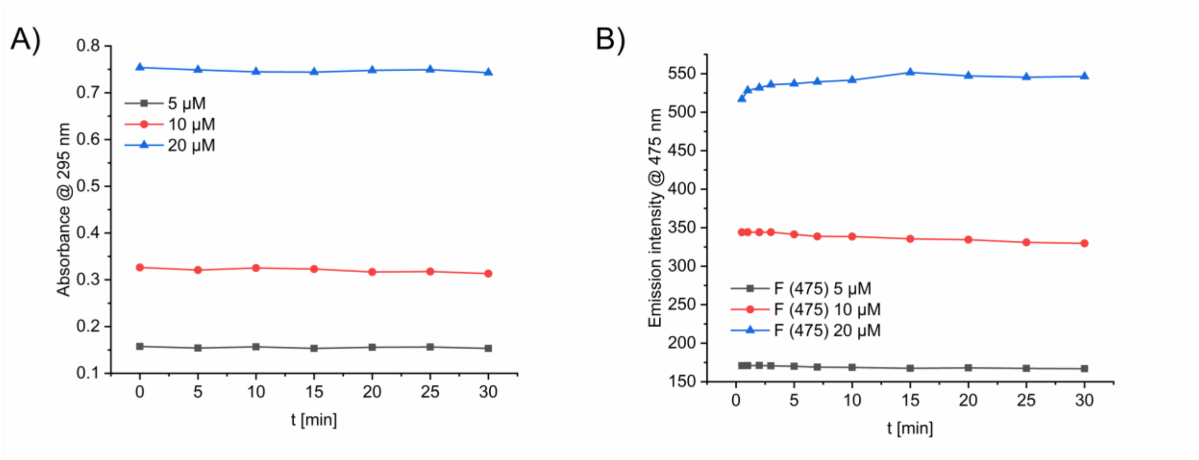

Figure S23: A) Time dependent UV–vis and B) emission intensity of ligand **1** in buffer solution at 5, 10 and 20 µM within 30 min.

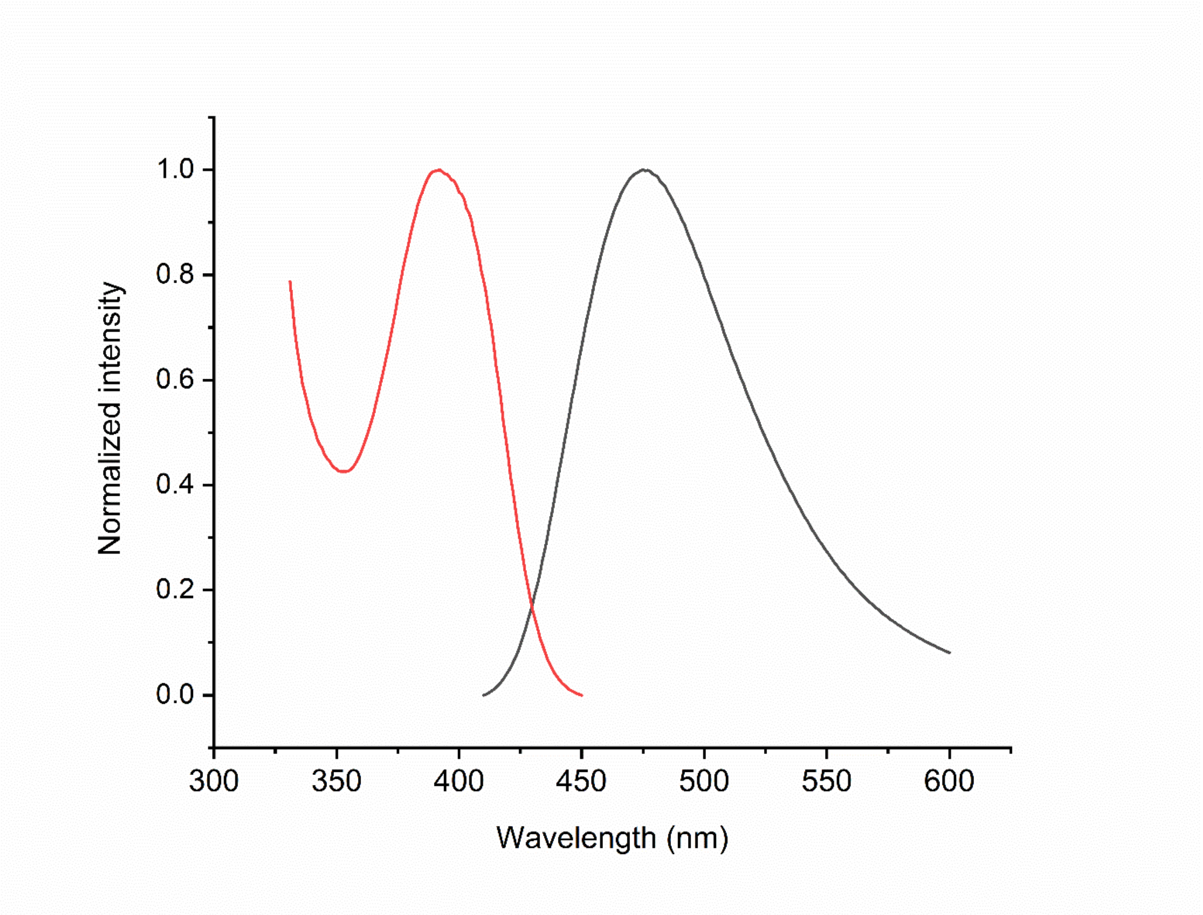

Figure S24: Normalized excitation and emissions spectrum of ligand **1** in buffer solution. Measurements were performed with an excitation wavelength of ≈390 nm and an emission wavelength of ≈470 nm.

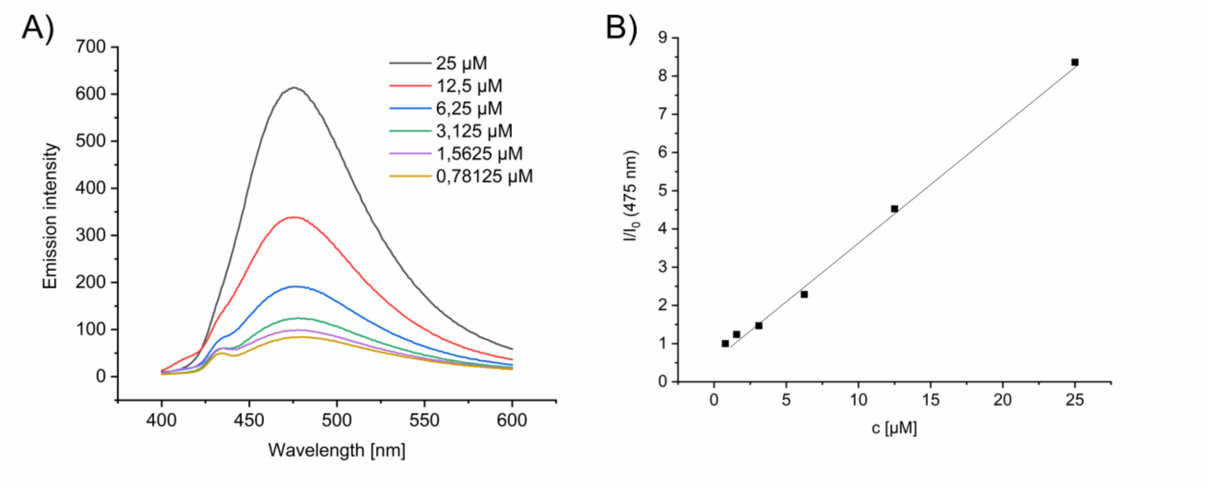

Figure S25: Fluorescence dilution series of ligand **1** in buffer solution with a linear behavior. Excitation wavelength ≈390 nm.

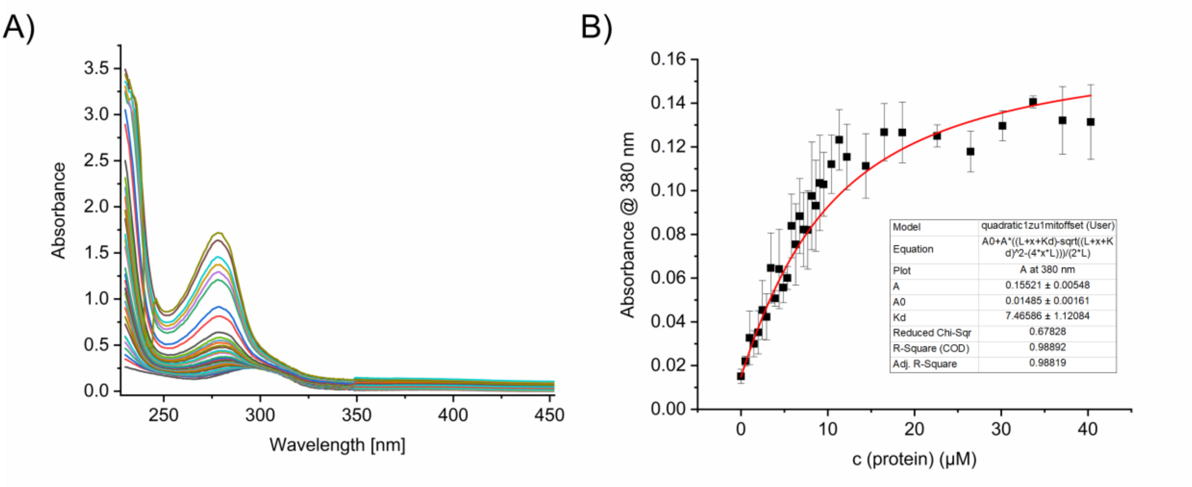

Figure S26: A) UV–vis- Titration of 14-3-3ζ protein to ligand **1** in buffer solution and B) determination of K_d via UV–vis titration of the 14-3-3ζ protein to ligand **1** at 380 nm in buffer solution.

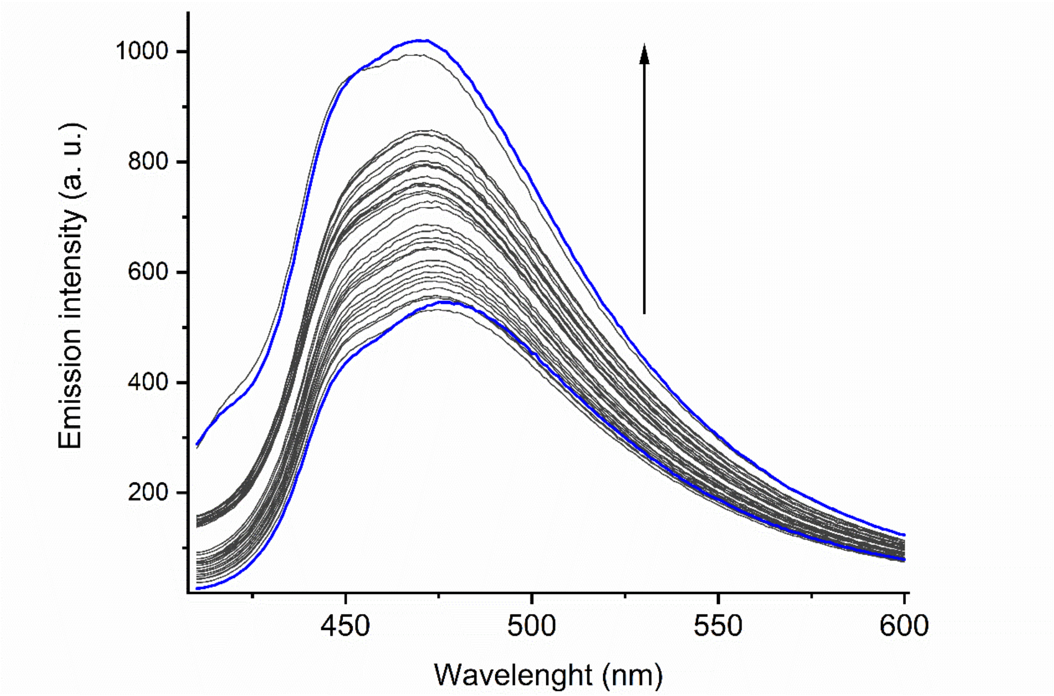

Figure S27: Fluorescence titration of 14-3-3ζ to **1**. The fluorescence signal is increasing with the addition of protein (0–8 equiv). Concentration **1** = 5 µM.

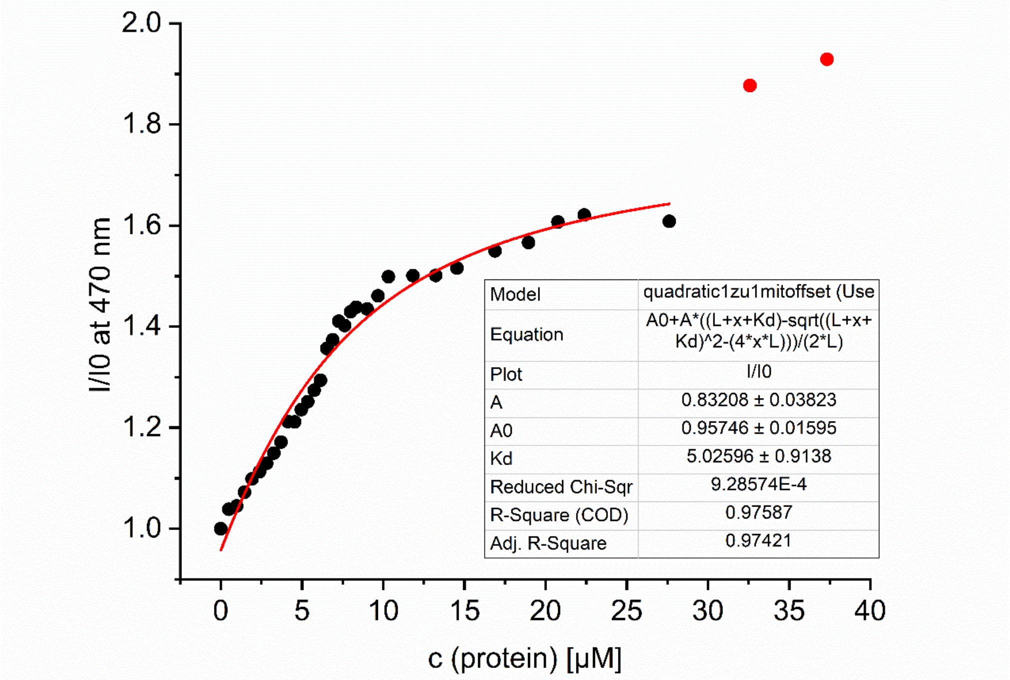

Figure S28: Fluorescence titration of 14-3-3ζ to **1** at 470 nm. Concentration of the ligand **1** was constant 5 µM. The K_d of the 1:1 complex was determined to be 5 ± 0.9 µM. The red data points are showing the start of the precipitation and were not considered for the fitting.

A Job's plot analysis was performed for more information about the ratio between ligand **1** and the 14-3-3ζ monomer [11]. The total concentration used of ligand **1** was 10 µM. The same conditions (buffer, pH value, temperature) as for the fluorescence titration were chosen.

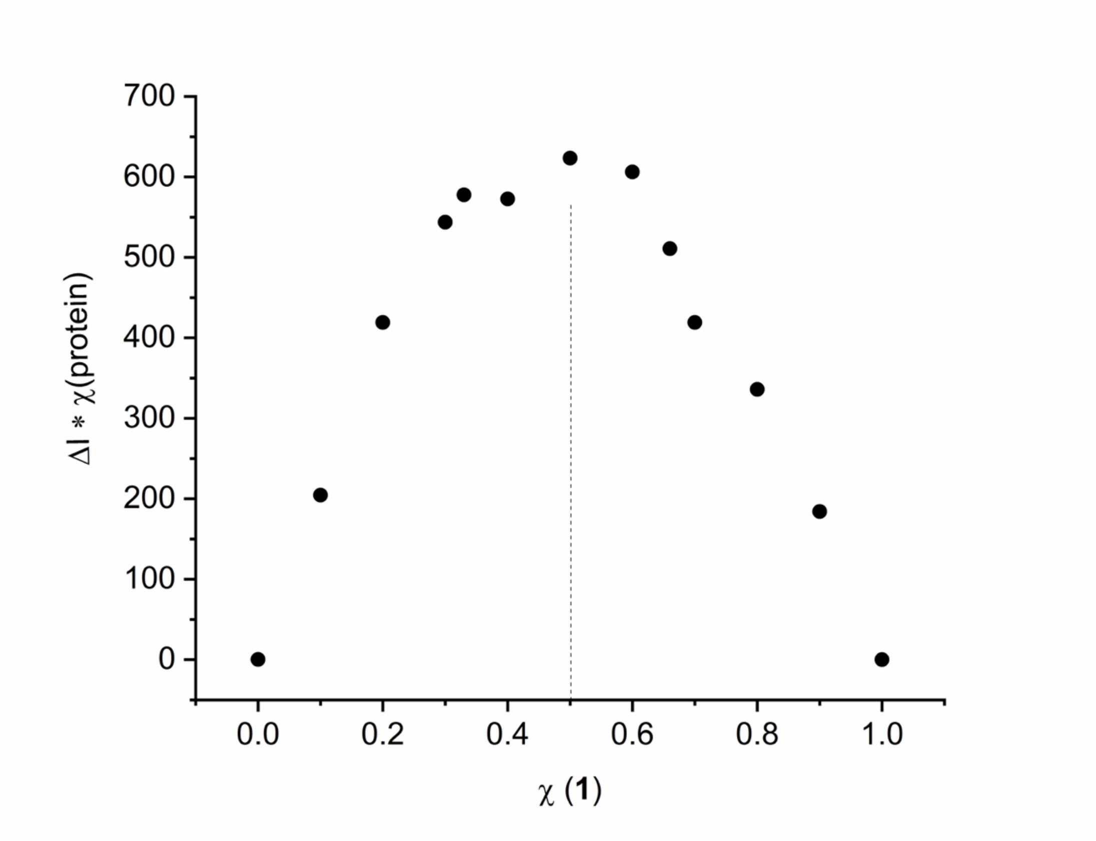

Figure S29: Job's plot of 14-3-3ζ and ligand **1** at λ_{em} = 470 nm. The maximum of 0.5 suggest a ratio of 1 ligand to 1 protein monomer or two ligands to a 14-3-3ζ dimer.

References

1. Grad, J.N.; Gigante, A.; Wilms, C.; Dybowski, J.N.; Ohl, L.; Ottmann, C.; Schmuck, C.; Hoffmann, D. *J. Chem. Inf. Model.* **2018,** *58*, 315-327.
2. Dodge, Y. *Statistical data analysis based on the L1-norm and related methods*; Birkhäuser, **2012**.
3. R 3.5.3; R Foundation for Statistical Computing: Vienna, AT, **2019**.

4. Riebe S.; Vallet C.; van der Vight F.; Gonzalez-Abradelo D.; Wölper C.; Strassert C. A.; Jansen G.; Knauer S.; Voskuhl J. *Chem.—Eur. J.* **2017,** *23*, 13660-13668.
5. Maity D.; Li M.; Ehlers M.; Schmuck C. *Chem. Commun.* **2017,** *53*, 208-211.
6. Choi, H.; Shirley, H.J.; Hume, P.A.; Brimble, M.A.; Furkert, D.P. *Angew. Chem., Int. Ed.* **2017,** *56*, 7420-7424.
7. Gottlieb, H. E.; Kotlyar V.; Nudelman, A. *J. Org. Chem.* **1997,** *62*, 7512-7515.
8. Fulmer, G. R.; Miller, A. J. M.; Sherden, N. H.; Gottlieb, H. E.; Nudelman, A.; Stoltz, B. M.; Bercaw, J. E.; Goldberg, K. I. *Organometallics* **2010,** *29*, 2176-2179.
9. Bayer, E.; Goettsch, S.; Mueller, J. W.; Griewel, B.; Guiberman, E.; Mayr, L. M.; P. Bayer. *J. Biol. Chem.* **2003,** *278,* No. 28, 26183 – 26193.
10. Mueller, B.; Restle, T.; Reinstein, J.; Goody, R.S. *Biochemistry* **1991,** *30,* 3709-3715.
11. Schalley, C. A. *Analytical Methods in Supramolecular Chemistry*, 2nd ed.; Wiley-VCH, 2012.
